# Venlafaxine and L-Thyroxine Treatment Combination: Impact on Metabolic and Synaptic Plasticity Changes in an Animal Model of Coexisting Depression and Hypothyroidism

**DOI:** 10.3390/cells10061394

**Published:** 2021-06-05

**Authors:** Katarzyna Głombik, Jan Detka, Bogusława Budziszewska

**Affiliations:** 1 Laboratory of Immunoendocrinology, Department of Experimental Neuroendocrinology, Maj Institute of Pharmacology, Polish Academy of Sciences, Smętna 12, 31-343 Kraków, Poland; detka@if-pan.krakow.pl (J.D.); budzisz@if-pan.krakow.pl (B.B.); 2 Department of Biochemical Toxicology, Medical College, Jagiellonian University, Medyczna 9, 30-688 Kraków, Poland

**Keywords:** depression, hypothyroidism, animal disease models, levothyroxine, venlafaxine, glycolysis, oxidative phosphorylation, mitochondria, brain

## Abstract

The clinical effectiveness of supportive therapy with thyroid hormones in drug-resistant depression is well-known; however, the mechanisms of action of these hormones in the adult brain have not been fully elucidated to date. We determined the effects of venlafaxine and/or L-thyroxine on metabolic parameters and markers involved in the regulation of synaptic plasticity and cell damage in an animal model of coexisting depression and hypothyroidism, namely, Wistar Kyoto rats treated with propylthiouracil. In this model, in relation to the depression model itself, the glycolysis process in the brain was weakened, and a reduction in pyruvate dehydrogenase in the frontal cortex was normalized only by the combined treatment with L-thyroxine and venlafaxine, whereas changes in pyruvate and lactate levels were affected by all applied therapies. None of the drugs improved the decrease in the expression of mitochondrial respiratory chain enzymes. No intensification of glucocorticoid action was shown, while an unfavorable change caused by the lack of thyroid hormones was an increase in the caspase-1 level, which was not reversed by venlafaxine alone. The results indicated that the combined administration of drugs was more effective in normalizing glycolysis and the transition to the Krebs cycle than the use of venlafaxine or L-thyroxine alone.

## 1. Introduction

Major depression, one of the most common mental illnesses, affects more individuals than all other psychiatric illnesses combined, and a significant number of patients do not remit after pharmacological or behavioral treatment. The low efficiency of the treatment of depression may be due to the poorly understood pathogenesis of this disease and the fact that most of the currently used drugs mainly affect serotonergic and noradrenergic transmission; while it is known that in this disease, metabolic, endocrine, and immune changes also occur [1,2,3,4]. In studies of the pathogenesis of depression, the focus has long been on disturbances in monoaminergic neurotransmission and the function of neurons [5]. However, as indicated by current studies, the disturbances of neurotransmission observed in depression may not be the primary cause but rather the consequence of metabolic alternations related mainly to the dysfunction of astrocytes, excessive action of pro-inflammatory cytokines released primarily from microglial cells [6,7], and the strong action of glutamate and glucocorticoids [8,9].

Currently, an increasing number of studies indicate that metabolic dysfunction and insufficient energy supply to neurons may be important in the pathogenesis of depression [10,11,12]. Glucose is the main source of energy for brain cells, and its metabolism provides energy for normal neuronal function, appropriate synthesis and function of neurotransmitters, and the formation of NADPH required for the removal of free radicals. In peripheral tissues, metabolism is regulated by some hormones, mainly insulin and thyroid hormones, as well as glucocorticoids and adrenaline. In contrast to peripheral tissues, the regulation of glucose metabolism and the role of particular cells of the central nervous system (CNS) in metabolic processes need further evaluation. Among the hormones whose altered level or action can lead to metabolic changes in the brain, thyroid hormones are a great matter of interest [13,14]. First, it has been shown that in the cases of drug-resistant and bipolar depression, in particular bipolar disorder with rapid phase change, supportive therapy with thyroid hormones enhances the effectiveness of antidepressants [15]. Although such therapy has been carried out for many years and its effectiveness has been well proven in the clinic, the impact of thyroid hormones on the function of the CNS in adults is insufficiently known. This is because the effects of thyroid hormones on the growth, differentiation, migration, and integration of neurons, glial proliferation, myelination, and neurotransmitter synthesis during development were established, but for a long time it was considered that after this period, genes regulated by thyroid hormones are insensitive to those hormones in the adult brain [16]. However, improvements in basic research methods have changed this view over time, and it is now known that thyroid hormones are also needed for the proper functioning of the CNS in adulthood [17,18]. Moreover, mental symptoms of depression and hypothyroidism are very similar, and in the clinic, hypothyroidism must be ruled out before depression is diagnosed. Epidemiologic data indicate that in patients with affective disorders, 1–4% hypothyroidism and 4–40% subclinical hypothyroidism occur [19]. Additionally, abnormalities of the T4 to T3 ratio, elevated rT3 levels, blunted TSH response to TRH, and the presence of anti-thyroid antibodies are reported more often in these patients than in the general population [13]. Although most people suffering from depression do not exhibit hypothyroidism, some studies suggest that in this disease, a reduction in the level or activity of thyroid hormones only within the brain can be observed [20]. Such central resistance to the action of thyroid hormones is supported by clinical data indicating a low incidence of adverse effects in depressed patients treated with supraphysiologic doses of thyroxine, in contrast to those observed in healthy people or with primary thyroid hypofunction [21]. The existence of differences in the levels of the active thyroid hormone triiodothyronine (T3) between the brain and blood is likely to occur because the T3 concentration in the brain depends on not only thyroid hormone synthesis in the thyroid gland but also, to a great extent, the expression of transporters and deiodinase enzymes in particular brain structures [22]. In line with this idea, in previous studies, we have shown a lower concentration of T3 in the frontal cortex of Wistar Kyoto rats (WKY), which are a model of endogenous depression, than in control Wistar rats, although the levels of this hormone in the blood did not differ significantly between the studied strains, and even in WKY rats, T3 levels in the serum showed a rising trend [23]. When determining the metabolic parameters in the examined depression model and in the animals with hypothyroidism, we observed changes in the markers of glycolysis in both studied models, while disturbances in mitochondrial respiration were only observed in the hypothyroidic rats. Moreover, in models of depression and hypothyroidism, we demonstrated similar changes in synaptic plasticity, e.g., a reduction in long-term potentiation in the dentate gyrus of the hippocampus and a decrease in short-term synaptic plasticity in the CA1 region of the hippocampus [24].

To date, the therapeutic efficacy of thyroxine in the adjunctive treatment of depression has been explained mainly by its effect on the intensification of serotonergic transmission or modulation of the β-adrenergic receptor [15,25]; however, the effect of this hormone on dysregulated metabolic processes in the brain observed in depression has not been taken into account or studied. Therefore, the goal of the current study was to determine the effect of levothyroxine (L-T4), administered alone and with an antidepressant drug, on selected metabolic parameters and markers related to the regulation of synaptic plasticity in an animal model of coexisting depression and hypothyroidism. WKY rats were used as a model of depression because it is a strain that exhibits hormonal and behavioral changes similar to those seen in depression, including increased stress reactivity [26,27]. Additionally, in this rat strain, considered a model of depression resistant to antidepressants, we conducted previous research on brain metabolism and synaptic plasticity [24,28]. Further, consistent with the frequent comorbidity of depression and anxiety disorders in humans, the WKY rats are a good model because, in addition to depressive-like behavior, they exhibit the anxiety-like behavior [27]. Moreover, as in the case of depression, association of hypothyroidism with anxiety disorders has also been shown [29]. To obtain a model of coexisting depression and hypothyroidism, WKY rats were treated with the anti-thyroid agent propylthiouracil (PTU) for three weeks. The antidepressant drug venlafaxine was chosen because it is now often used as a selective norepinephrine and serotonin reuptake inhibitor, and the interaction of thyroid hormones with both of these neurotransmitters was observed [15]. The effect of thyroid hormone (L-T4), given alone or with venlafaxine, in a rat model of coexisting depression with anxiety and hypothyroidism on selected metabolic markers was determined based on our previous research. Among the metabolic processes, we paid special attention to the mechanism of astrocyte-neuron lactate shuttling. During periods of impaired glucose availability or increased neurons energy requirements, astrocytes glycolytically metabolize glucose or glycogen to lactate, which is shuttled to the neurons via the extracellular fluid. Current research indicates that this process of lactate production in astrocytes and its subsequent transport to neurons is disturbed in neurodegenerative and mental diseases, including depression [30]. Lactate, in addition to its role in the glial–neuronal metabolic linkage, also plays a neuromodulatory role acting through the G-protein-coupled receptor for lactate (HCAR1; GPR81). Lactate has been shown to regulate neuronal function, including excitability, plasticity, and memory consolidation and, moreover, its antidepressant effect was observed in mice [31]. In addition to changes in metabolic markers, due to the neuroprotective effects of thyroid hormones, we also assessed the effects of hypothyroidism and applied therapies on selected factors important in cell damage processes and the potential contribution of glucocorticoids or glutamate.

## 2. Materials and Methods

### 2.1. Animals

All experimental procedures were carried out in accordance with the Local Ethics Committee, Kraków, Poland (permit No. 46/2018 of 01.02.2018). Male Wistar Kyoto rats (*n* = 60, aged 8 weeks upon arrival) were obtained from Charles River Laboratories (Charles River Laboratories, Hamburg, Germany) and were maintained under standard conditions (room temperature of 23 °C, 12/12-h light/dark cycle, lights on at 6:00 a.m.).

### 2.2. Experimental Design

**Figure 1:**
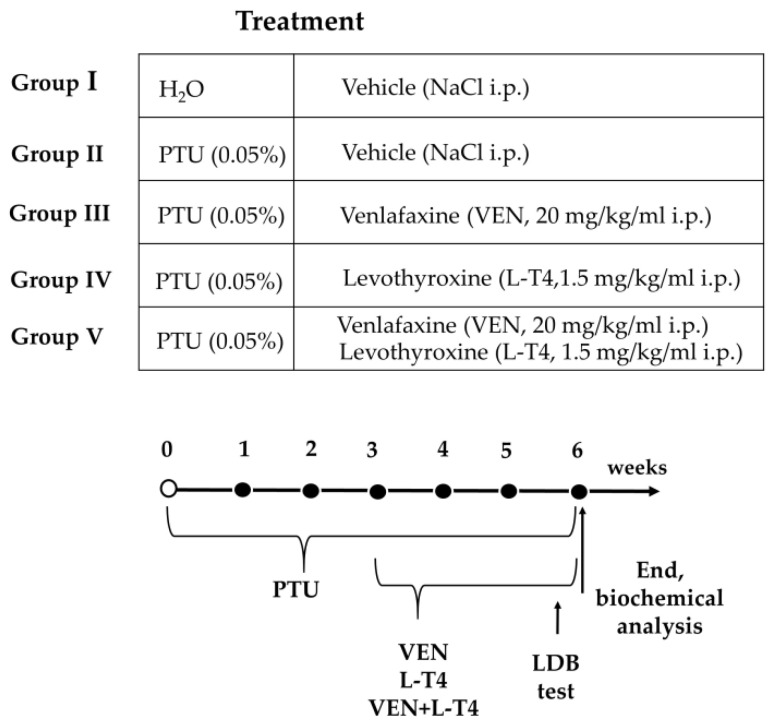
Scheme of the experimental design.

After acclimation, the animals were randomly divided into five groups: group I: Wistar Kyoto rats injected with 0.9% NaCl i.p. for the last three weeks of the experiment; group II: Wistar Kyoto rats treated with 0.05% *w*/*v* PTU in their drinking water for six weeks and injected with 0.9% NaCl i.p. for the last three weeks of the experiment, group III: Wistar Kyoto rats treated with 0.05% *w*/*v* PTU in their drinking water for six weeks and with venlafaxine (VEN, 20 mg/kg/mL dissolved in 0.9% NaCl i.p.) for the last three weeks of the experiment, group IV: Wistar Kyoto rats treated with 0.05% *w*/*v* PTU in their drinking water for six weeks and with levothyroxine (L-T4, 1.5 mg/kg/mL dissolved in 0.9% NaCl i.p.) for the last three weeks of the experiment, group V: Wistar Kyoto rats treated with 0.05% *w*/*v* PTU in their drinking water for six weeks and with VEN (20 mg/kg/mL dissolved in 0.9% NaCl i.p.) and L-T4 (1.5 mg/kg/mL dissolved in 0.9% NaCl i.p.) for the last three weeks of the experiment. The timeline showing the experimental design is illustrated in Figure 1.

### 2.3. Chemicals and Drugs

The following agents and drugs were used: propylthiouracil (PTU; 0.05%; Sigma-Aldrich, Saint Louis, MO, USA), venlafaxine (VEN, 20 mg/kg/mL; Carbosynth, Compton, UK), and levothyroxine sodium salt pentahydrate (L-T4, equal to 1.5 mg/kg/mL levothyroxine; Sigma-Aldrich, Saint Louis, MO, USA).

### 2.4. Behavioral Testing—Light-Dark Box (LDB) Test

In the last three days of the experiment, the animals underwent a light-dark box test (LDB). The light dark-box test was carried out according to the procedure described by Chocyk et al. [32] and Chamera et al. [33] in an apparatus consisting of a cage divided into two sections of equal size (one chamber was brightly illuminated by diodes (100 lux), whereas the other chamber was dark) by a partition with a door (10.6 cm × 10.4 cm). The apparatus was controlled with a computer system (TSE Systems, Bad Homburg, Germany). The whole experiment was carried out in a dark room, and the rats were kept in darkness for an hour before the test. At the beginning of each test session, the animal was placed in one corner of the light compartment, facing away from the gate. Then, the animal was allowed to move freely between the two chambers with the door open for 10 min. The behavioral response of rats during the trials was recorded by Fear Conditioning Software (TSE, Bad Homburg, Germany). The time spent in each compartment was recorded for each animal. After each trial, the chambers were cleaned to prevent bias based on olfactory cues.

### 2.5. Biochemical Analysis

The rats were sacrificed under nonstressed conditions by rapid decapitation. Trunk blood samples were collected into plastic test tubes containing EDTA as an anticoagulant. After centrifugation (3000 rpm, 20 min, 4 °C), separated plasma was gathered and frozen at −20 °C until use. Brain structures (frontal cortex and hippocampus) were dissected on ice-cold glass plates, frozen on dry ice, and stored at −80 °C until further analysis.

#### 2.5.1. Thyroid Hormone Measurement in Plasma

Plasma concentrations of free T3, free T4 (both: DiaMetra, Perugia, Spello, Italy), and TSH (Demeditec, Kiel, Germany) were measured with enzyme-linked immunosorbent assays (ELISAs) and are presented as pg/mL, ng/dL, and ng/mL, respectively.

#### 2.5.2. Glucose, Total Cholesterol, HDL, LDL, and Triglyceride Measurements in Plasma

Plasma glucose and lipid measurements were performed on a Mindray BS-800 Chemistry Analyzer (Mindray, Shenzhen, China). The concentrations of the measured factors are presented as mg/dL.

#### 2.5.3. Tissue Homogenate Preparation and Determination of Protein Concentration

Samples were homogenized in 2-mL plastic tubes filled with an appropriate buffer using a TissueLyser II (Qiagen Inc, Valencia, CA, USA) as described previously [34]. The protein level was measured using Pierce™ BCA Protein Assay Kit (Thermo Fisher Scientific, Rockford, IL, USA) [35].

#### 2.5.4. Preparation of Mitochondria-Enriched and Cytosolic Fractions

To measure the concentrations and activities of mitochondrial enzymes, mitochondria-enriched membrane fractions were isolated from the frontal cortex and hippocampus according to the procedure described by Wernicke [36]. Brain tissue samples were homogenized in buffer containing 5 mol/L HEPES/NaOH, pH 7.4, 320 mmol/L sucrose, 1 mmol/L Na+/EDTA, and 0.5% protease inhibitor cocktail (Thermo Fisher Scientific, Waltham, MA, USA) using a Teflon-glass homogenizer. Then, samples were centrifuged at 1300× *g* for 4 min at 4 °C, and supernatants were collected. The pellets were rinsed twice and centrifuged at 1500× *g* for 4 min at 4 °C. The combined supernatants were centrifuged at 17,000× *g* for 12 min at 4 °C, after which the mitochondria-containing pellets and supernatants (cytosolic fraction) were collected.

#### 2.5.5. Pyruvate Measurement

The level of pyruvate in the cytosolic fraction isolated from the brain structures was measured using a fluorimetric assay kit (BioVision, Milpitas, CA, USA). Samples and pyruvate standards in 96-well plates were incubated with reaction mix for 30 min, after which fluorescence was measured at Ex/Em = 535/590 in a fluorometer (Tecan Infinite M 1000, Männedorf, Switzerland). The concentration of pyruvate was calculated based on the standard curve and displayed as nmol of pyruvate per mg of protein.

#### 2.5.6. Pyruvate Dehydrogenase (PDH) Expression and Activity Estimation

Pyruvate dehydrogenase levels in the mitochondria-enriched fraction of the frontal cortex and hippocampus were estimated using an ELISA method (Shanghai Qayee Biotechnology Co., Shanghai, China) according to the manufacturer’s instructions. The absorbance was measured using a spectrophotometer (Tecan Infinite M200 Pro, Männedorf, Switzerland) at a wavelength of λ = 450. The concentration of PDH in the samples was calculated based on the standard curve and expressed as ng/mg protein.

PDH activity was measured with a colorimetric assay kit (BioVision, Milpitas, CA, USA) in a spectrophotometer (Tecan Infinite M200 Pro, Switzerland) according to the manufacturer’s instructions. The absorbance was measured at 37 °C immediately after starting the reaction and after 1 h at a wavelength λ = 450 nm. The activity of PDH was calculated based on the standard curve and displayed as nanomoles per min per mg of protein.

#### 2.5.7. Lactate Measurement

The levels of lactate in the mitochondria-enriched and cytosolic fractions obtained from the frontal cortex and hippocampus were measured with a colorimetric assay kit (BioVision, Milpitas, CA, USA). Samples and standards were mixed with the reaction mix on a 96-well plate, and after a 30-min incubation, the absorbance was measured at λ = 570 nm using a spectrophotometer (Tecan Infinite M200 Pro, Männedorf, Switzerland). The concentration of lactate was calculated based on the standard curve and displayed as nmol/mg protein.

#### 2.5.8. Protein Levels of the OXPHOS Complexes, Monocarboxylate Transporters (MCT2, MCT4), Hydroxycarboxylic Acid Receptor (HCAR1), Glucocorticoid Receptor (GR), FK506-Binding Protein 51 (FKBP51), Bax, Beclin 1, and Caspase-1—Western Blot Analysis

The tissue homogenates and mitochondria-enriched fractions were prepared as previously described [23,24,37]. The obtained samples of tissue homogenates were homogenized in 2% SDS (BioShop, Burlington, ON, Canada) in dH_2_O, whereas samples of isolated mitochondria-enriched membrane fraction were prepared through lysis in PBS containing 1% Triton X-100 and 0.1% SDS with phosphate and protease inhibitors (Thermo Fisher Scientific, Waltham, MA, USA). Equal amounts of protein were separated on 20% (*w*/*v*) Criterion™ TGX™ Precast Midi Protein Gels (Bio-Rad, Hercules, CA, USA) and transferred onto PVDF membranes (Sigma-Aldrich, Saint Louis, MO, USA). After semidry transfer (though for OXPHOS, wet transfer was conducted in CAPS buffer), some membranes were cut to allow simultaneous incubation with different antibodies. Then, membranes were blocked in 5% skim milk in Tris-buffered saline (TBS) with 0.05% Tween 20 (Sigma-Aldrich, Saint Louis, MO, USA) for 1 h at room temperature (RT) and were incubated overnight at 4 °C with primary antibodies against the following proteins: Total OXPHOS Rodent WB Antibody Cocktail (Abcam, Cambridge, UK), MCT2, MCT4 (both Bioassay Technology Laboratory, Shanghai, China), HCAR1 (Sigma-Aldrich, Saint Louis, MO, USA), FKBP51, Bax (both ProteinTech, Manchester, UK), GR, beclin 1, and caspase-1 (Santa Cruz Biotechnology, Dallas, TX, USA). The next day, the membranes were extensively washed in TBST (TBS with 0.05% Tween 20) and incubated at RT with horse anti-mouse/goat anti-rabbit IgG HRP peroxidase-conjugated secondary antibody (Vector Laboratories, Peterborough, UK) for 1 h. After washing in TBST (Tris-buffered saline with 0.05% Tween 20), the bands were detected using BM Chemiluminescence Western Blotting Substrate (POD) (Roche, Mannheim, Germany) in a luminescent image analyzer with a Fujifilm LAS-1000 System (Fujifilm, Tokyo, Japan). The relative levels of immunoreactivity were densitometrically quantified using Fujifilm Multi Gauge software (Fujifilm, Tokyo, Japan). As an internal loading control, the amount of vinculin (Sigma-Aldrich, Saint Louis, MO, USA) was determined. Some membranes were stripped and reprobed.

#### 2.5.9. OPA1, DNM1L, GLT-1, GLAST Protein Levels

The levels of OPA1, DNM1L (both Bioassay Technology Laboratory, Shanghai, China), GLT-1, and GLAST (both ELK Biotechnology, Wuhan, China) in the tissue homogenates extracted from the frontal cortex and hippocampus in PBS buffer were estimated using commercially available ELISA kits. The absorbance was measured using a Tecan Infinite M200 Pro (TECAN, Männedorf, Switzerland) set to the wavelength λ = 450 nm. The concentration of measured protein in the samples was calculated based on the relevant standard curve and expressed as ng/mg protein.

### 2.6. Statistical Analysis

All graphs were prepared using GraphPad Prism 8 (San Diego, CA, USA). The results of the experiments are displayed as the means ± SEM. Statistical analysis was performed using Statistica 13.3 software (StatSoft, Palo Alto, CA, USA). The differences between WKY + PTU vs. WKY (control); WKY + PTU + VEN vs. WKY (control), WKY + PTU + L-T4 vs. WKY (control), and WKY + PTU + VEN + L-T4 vs. WKY (control) were analyzed by one-way analyses of variance (ANOVA), whereas WKY + PTU + VEN, WKY + PTU + L-T4, and WKY + PTU + VEN + L-T4 vs. WKY + PTU were analyzed by factorial ANOVA followed by the Duncan post hoc test when appropriate. Differences were considered statistically significant when the determined *p*-value was less than 0.05 (*p* < 0.05). The ANOVA results are reported as an F-statistic and its associated degrees of freedom.

## 3. Results

### 3.1. Light-Dark Box Test

**Figure 2:**
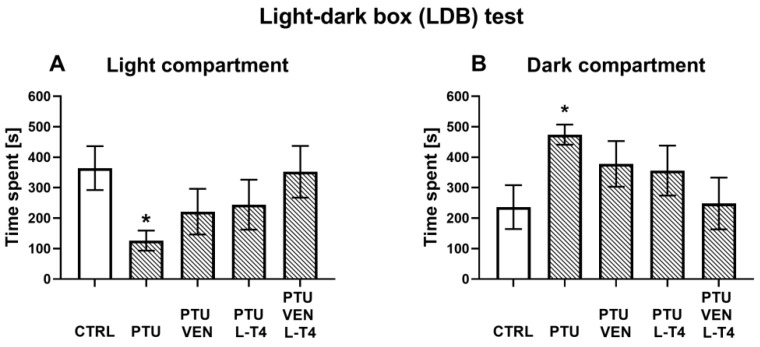
Effects of propylthiouracil (PTU) in Wistar Kyoto (WKY) rats and venlafaxine (VEN), levothyroxine (L-T4), and VEN + L-T4 administration on time spent in the light compartment (**A**) and the dark compartment (**B**) measured in the light-dark box test in PTU-treated WKY rats. The results are expressed as the mean ± SEM. * *p* < 0.05 vs. the WKY (control group). *n* = 8–10. Statistics: one-way/factorial analysis of variance (ANOVA), post hoc comparison: Duncan test, when appropriate.

The light-dark box test was used to examine anxiety-like behavior, which is often observed during depression. The WKY rats treated with PTU were characterized by a decrease in the time spent in the light compartment (F_1,15_ = 9.84, *p* < 0.05) and by an increase in the time spent in the dark compartment (F_1,15_ = 8.09, *p* < 0.05) when compared to the WKY control rats. Applied drugs did not significantly regulate anxiety-like behavior in comparison to the WKY PTU-treated group (Figure 2A,B).

### 3.2. Thyroid Hormone Plasma Concentrations

**Figure 3:**
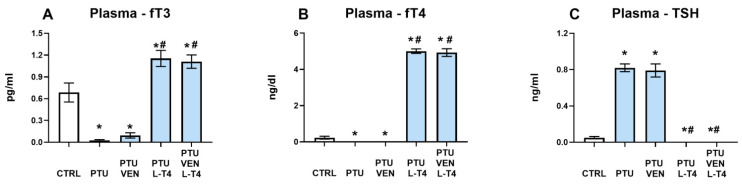
Effects of propylthiouracil (PTU) in Wistar Kyoto (WKY) rats and venlafaxine (VEN), levothyroxine (L-T4), and VEN + L-T4 administration on fT3 (**A**), fT4 (**B**), and TSH (**C**) levels in plasma in PTU-treated WKY rats. The results are expressed as the mean ± SEM. * *p* < 0.05 vs. the WKY (control group); # *p* < 0.05 vs. the WKY + PTU (coexisting depression and hypothyroidism group). *n* = 9–12. Statistics: one-way/factorial analysis of variance (ANOVA), post hoc comparison: Duncan test, when appropriate.

The fT3 level was diminished by PTU treatment (PTU effect: F_1,20_ = 20.98, *p* < 0.05), whereas additional administration of L-T4 (L-T4 effect: F_1,42_ = 189.31, *p* < 0.05) resulted in significant increases in fT3 in both treated groups (alone and combined with VEN). PTU + VEN-treated animals did not differ from PTU-treated animals (Figure 3A).

Estimation of fT4 in the plasma showed similar effects as in fT3 measurements. PTU significantly downregulated the concentration of fT4 in the control group (PTU effect: F_1,19_ = 10.62, *p* < 0.05). L-T4 led to increases in fT4 in the WKY + PTU+ L-T4 and WKY + PTU + VEN + L-T4 groups (L-T4 effect: F_1,44_ = 1615.30, *p* < 0.05). VEN administration alone did not modulate the PTU effect (Figure 3B).

The TSH level in the serum was elevated by PTU (PTU effect: F_1,22_ = 298.00, *p* < 0.05). Treatment with L-T4 in PTU animals led to significant reductions in the level of this hormone in both groups: L-T4 alone and L-T4 + VEN (L-T4 effect: F_1,44_ = 369.81, *p* < 0.05). Treatment with VEN alone did not influence this change (Figure 3C).

### 3.3. Glucose Level and Lipid Panel (Total Cholesterol, HDL, LDL, Triglyceride) in Plasma

**Figure 4:**
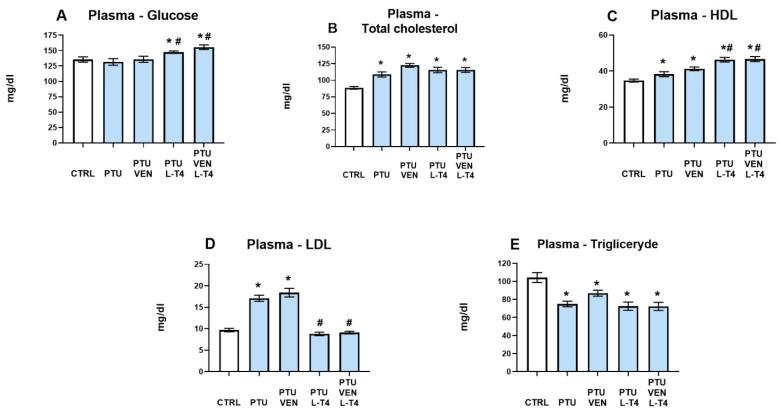
Effects of propylthiouracil (PTU) in Wistar Kyoto (WKY) rats and venlafaxine (VEN), levothyroxine (L-T4), and VEN + L-T4 administration on glucose (**A**), total cholesterol (**B**), HDL (**C**), LDL (**D**), and triglyceride (**E**) levels measured in the plasma of PTU-treated WKY rats. The results are expressed as the mean ± SEM. * *p* < 0.05 vs. the WKY (control group); # *p* < 0.05 vs. the WKY + PTU (coexisting depression and hypothyroidism group). *n* = 10. Statistics: one-way/factorial analysis of variance (ANOVA), post hoc comparison: Duncan test, when appropriate.

The level of glucose in the plasma was increased by L-T4 alone or with VEN in PTU-treated animals (L-T4 effect: F_1,36_ = 17.76, *p* < 0.05) and in comparison to the control animals (PTU + L-T4 effect: F_1,18_ = 6.38; PTU + VEN + L-T4 effect: F_1,18_ = 12.44, *p* < 0.05) (Figure 4A).

The total cholesterol level was elevated by PTU (PTU effect: F_1,18_ = 20.27, *p* < 0.05), and treatment with drugs alone or in combination did not affect the PTU-induced effect (Figure 4B).

Additionally, PTU treatment led to an elevation in HDL levels (PTU effect: F_1,18_ = 5.11, *p* < 0.05). Treatment with L-T4 alone and together with VEN intensified the PTU-induced effect (L-T4 effect: F_1,36_ = 28.50, *p* < 0.05) (Figure 4C).

A significant enhancing effect of PTU was observed in the case of LDL (PTU effect: F_1,18_ = 94.39, *p* < 0.05). L-T4 alone and together with VEN reversed the PTU-induced elevation in LDL (L-T4 effect: F_1,36_ = 183.54, *p* < 0.05). Treatment with VEN alone did not influence the change evoked by PTU (Figure 4D).

PTU also significantly diminished the level of plasma triglyceride (PTU effect: F_1,18_ = 21.48, *p* < 0.05), which was not normalized by VEN and/or L-T4 administration (Figure 4E).

### 3.4. Pyruvate Level in the Brain

**Figure 5:**
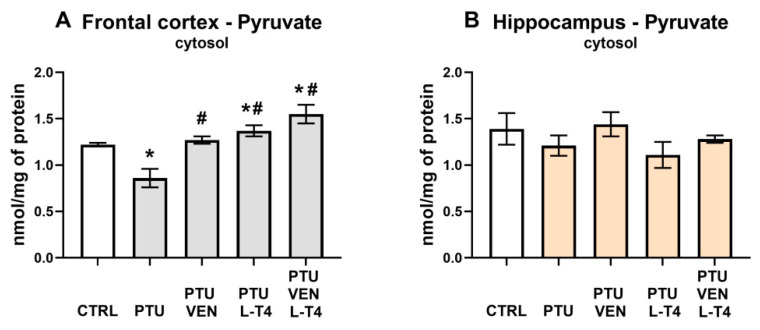
Effects of propylthiouracil (PTU) in Wistar Kyoto (WKY) rats and venlafaxine (VEN), levothyroxine (L-T4), and VEN + L-T4 administration on pyruvate levels in the cytosolic fraction of the frontal cortex (**A**) and the hippocampus (**B**) in PTU-treated WKY rats. The results are expressed as the mean ± SEM. * *p* < 0.05 vs. the WKY (control group); # *p* < 0.05 vs. the WKY + PTU (coexisting depression and hypothyroidism group). *n* = 7–8. Statistics: one-way/factorial analysis of variance (ANOVA), post hoc comparison: Duncan test, when appropriate.

In the cytosolic fraction of the frontal cortex, the pyruvate concentration was reduced in PTU-treated animals (PTU effect: F_1,13_ = 14.78, *p* < 0.05). Both VEN and L-T4 reversed the PTU-induced effect (VEN effect: F_1,27_ = 13.84, *p* < 0.05; L-T4 effect: F_1,27_ = 24.34), but no interaction was observed. Additionally, the PTU + L-T4-treated and PTU + VEN + LT-4-treated groups showed greater levels of pyruvate than the control WKY group (PTU + L-T4 effect: F_1,14_ = 4.82, *p* < 0.05, PTU + VEN + L-T4 effect: F_1,14_ = 9.51, *p* < 0.05) (Figure 5A). No changes in the hippocampus were detected (Figure 5B).

### 3.5. Concentration and Activity of PDH in the Brain

**Figure 6:**
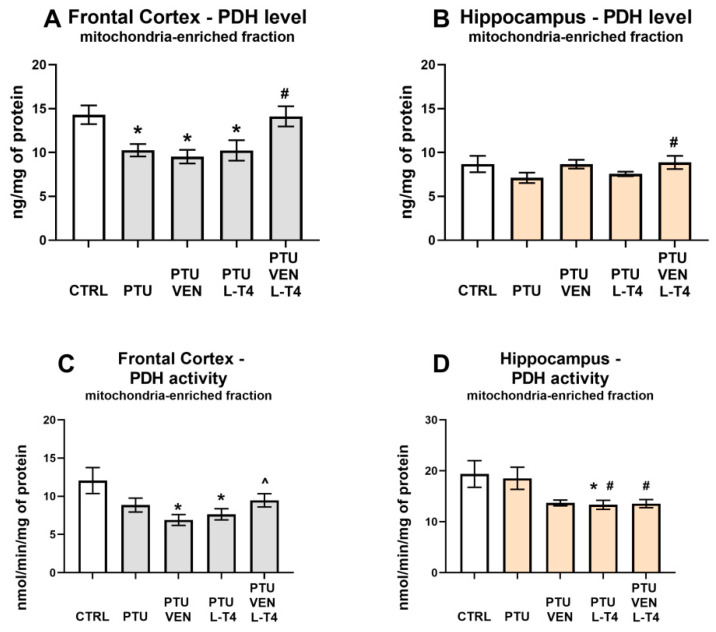
Effects of propylthiouracil (PTU) in Wistar Kyoto (WKY) rats and venlafaxine (VEN), levothyroxine (L-T4), and VEN + L-T4 administration on pyruvate dehydrogenase (PDH) levels and activity in the mitochondria-enriched fraction of the frontal cortex (**A**,**C**) and the hippocampus (**B**,**D**) in PTU-treated WKY rats. The results are expressed as the mean ± SEM. * *p* < 0.05 vs. the WKY (control group); # *p* < 0.05 vs. the WKY + PTU (coexisting depression and hypothyroidism group); ^ vs. the WKU + PTU + VEN group. *n* = 8–10. Statistics: one-way/factorial analysis of variance (ANOVA), post hoc comparison: Duncan test, when appropriate.

In the frontal cortex, PTU administration diminished the level of PDH (PTU effect: F_1,14_ = 8.46, *p* < 0.05), while VEN and L-T4 co-treatment reversed the PTU-induced effect (VEN x L-T4 interaction: F_1,33_ = 5.03, *p* < 0.05). Treatment with VEN or L-T4 alone did not affect the PDH level in this brain structure in groups treated with PTU (Figure 6A). In the hippocampus, VEN in rats treated with PTU significantly upregulated the level of PDH (VEN effect: F_1,34_ = 6.50, *p* < 0.05) (Figure 6B).

Measurements of PDH activity in the frontal cortex revealed that animals treated with PTU and VEN or L-T4 displayed reduced activity of this enzyme vs. WKY animals (PTU VEN effect: F_1,14_ = 7.82, PTU L-T4 effect: F_1,14_ = 5.71, *p* < 0.05). In PTU-treated animals, an effect of co-treatment was also shown (VEN x L-T4 interaction: F_1,28_ = 5.47, *p* < 0.05); animals that underwent combined treatment showed an increase in the activity of PDH compared to animals treated only with VEN (Figure 6C). In the hippocampus, treatment with PTU did not change the activity of PDH; however, a decreased enzyme activity was observed in the PTU + L-T4 group in comparison to the control (PTU + L-T4 effect: F_1,14_ = 4.82, *p* < 0.05). Rats receiving PTU and drugs showed the impact of L-T4 (F_1,28_ = 4.36, *p* < 0.05) and reduced PDH activity was observed in the hippocampi of rats treated with L-T4 alone or with VEN (Figure 6D).

### 3.6. Lactate Level in the Brain

**Figure 7:**
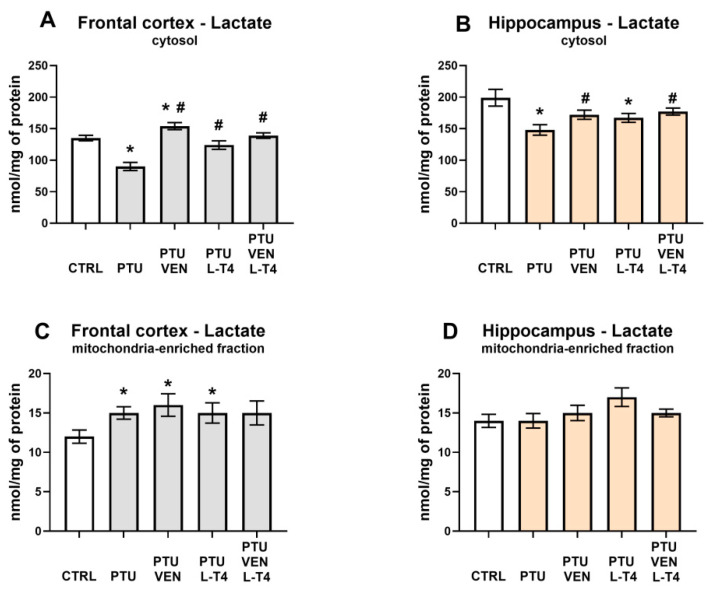
Effects of propylthiouracil (PTU) in Wistar Kyoto (WKY) rats and venlafaxine (VEN), levothyroxine (L-T4), and VEN + L-T4 administration on lactate levels in the cytosolic and mitochondria-enriched fraction of the frontal cortex (**A**,**C**) and the hippocampus (**B**,**D**) in PTU-treated WKY rats. The results are expressed as the mean ± SEM. * *p* < 0.05 vs. the WKY (control group); # *p* < 0.05 vs. the WKY + PTU (coexisting depression and hypothyroidism group). *n* = 7–8. Statistics: one-way/factorial analysis of variance (ANOVA), post hoc comparison: Duncan test, when appropriate.

In both examined brain areas, PTU decreased the levels of lactate in the cytosolic fraction (PTU effect: F_1,12_ = 35.52 in FCx, F_1,12_ = 10.86 in Hp; *p* < 0.05) (Figure 7A,B). Furthermore, in the frontal cortex administration of VEN, L-T4, and co-treatment with these drugs normalized the observed PTU-induced change (VEN × L-T4 interaction: F_1,26_ = 16.63, *p* < 0.05) (Figure 7A), whereas in the hippocampus, a normalization effect was observed only in the case of VEN (VEN effect: F_1,26_ = 5.96, *p* < 0.05, in groups treated with VEN and VEN + L-T4) (Figure 7B). In the frontal cortex, in WKY PTU rats treated with VEN alone, cytosolic lactate was increased in comparison to control WKY animals (F_1,14_ = 7.23, *p* < 0.05) (Figure 7A), whereas in the hippocampus in the WKY + PTU + L-T4 group, lactate was reduced, as in PTU-treated animals (Figure 7B).

Taking into account the mitochondria-enriched fraction in the frontal cortex, PTU treatment led to an elevation in lactate levels (PTU effect: F_1,13_ = 8.00, *p* < 0.05) (Figure 7C). This effect dependence was still present after VEN or L-T4 administration. In the hippocampus, no changes in the concentration of lactate in the mitochondria-enriched fraction were detected (Figure 7D).

### 3.7. Hydroxycarboxylic Acid Receptor 1 (HCAR1) Level in the Brain

There was no impact of either PTU or applied treatment on HCAR1 levels in the frontal cortex and hippocampus (Table 1).

### 3.8. Monocarboxylate Transporters MCT2 and MCT4 Levels in the Brain

There were no changes in the protein levels of MCT2 and MCT4 in either examined brain structure (Table 1).

### 3.9. Levels of Oxidative Phosphorylation (OXPHOS) Complexes in the Mitochondria-Enriched Fraction of the Brain

**Figure 8:**
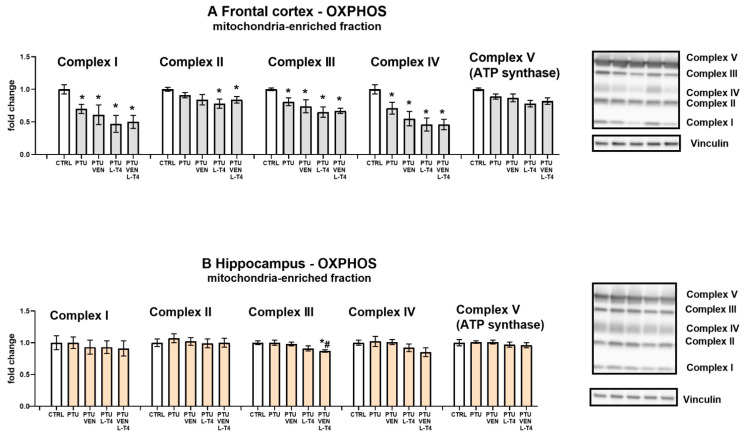
Effects of propylthiouracil (PTU) in Wistar Kyoto (WKY) rats and venlafaxine (VEN), levothyroxine (L-T4), and VEN + L-T4 administration on oxidative phosphorylation (OXPHOS) complexes in the mitochondria-enriched fraction of the frontal cortex (**A**) and the hippocampus (**B**) in PTU-treated WKY rats. The bands from the left: WKY, WKY + PTU, WKY + PTU + VEN, WKY + PTU + L-T4, and WKY + PTU + VEN + L-T4. The results are expressed as the mean ± SEM. * *p* < 0.05 vs. the WKY (control group); # *p* < 0.05 vs. the WKY + PTU (coexisting depression and hypothyroidism group). *n* = 8. Statistics: one-way/factorial analysis of variance (ANOVA), post hoc comparison: Duncan test, when appropriate (See Supplementary Materials).

In the frontal cortex in groups treated with PTU, significant reductions in the expression of oxidative phosphorylation complexes I, III, and IV were observed (PTU effect: F_1,14_ = 8.86, complex I; PTU effect: F_1,14_ = 9.73, complex III; PTU effect: F_1,14_ = 6.82, complex IV, *p* < 0.05). Injections with VEN and/or L-T4 did not modulate the impact of PTU on these proteins. In complex II, a PTU effect was not observed, but in the PTU + L-T4- and PTU + VEN + L-T4-treated groups, a reduction in the level of this factor was shown (Figure 8A). In both examined brain areas, the expression of complex V did not differ between the groups (Figure 8A,B). Furthermore, no changes in the hippocampus were demonstrated in the case of complexes I, II, and IV, but in complex III, an L-T4 effect was observed in PTU + VEN + L-T4 vs. PTU-treated animals (F_1,28_ = 8.88, *p* < 0.05). These animals had also diminished level of complex III in comparison to control WKY rats (PTU + VEN + L-T4 effect, F_1,14_ = 9.26).

### 3.10. Levels of Mitochondrial Fusion Factor, OPA-1, and Fission Factor, DNM1L

**Figure 9:**
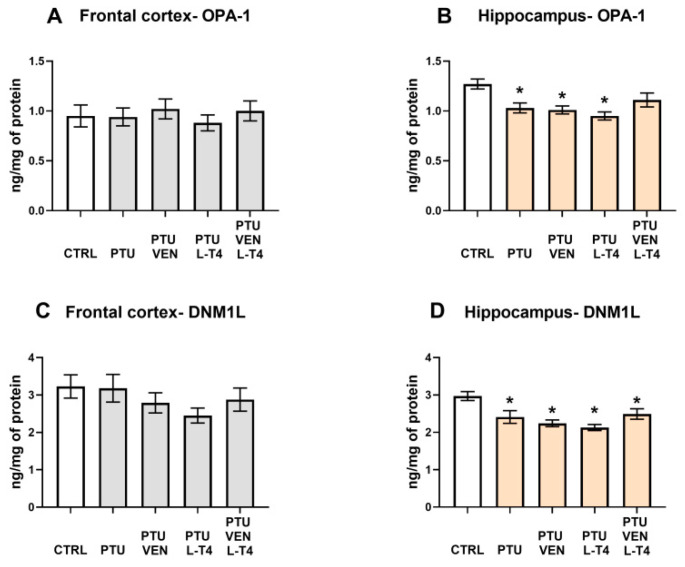
Effects of propylthiouracil (PTU) in Wistar Kyoto (WKY) rats and venlafaxine (VEN), levothyroxine (L-T4), and VEN + L-T4 administration on OPA-1 and DNML1 in tissue homogenates of the frontal cortex (**A**,**C**) and the hippocampus (**B**,**D**) in PTU-treated WKY rats. The results are expressed as the mean ± SEM. * *p* < 0.05 vs. the WKY (control group). *n* = 9–10. Statistics: one-way/factorial analysis of variance (ANOVA), post hoc comparison: Duncan test, when appropriate.

There were no significant changes in OPA-1 expression in the frontal cortices between the examined groups (Figure 9A). In the hippocampi of WKY PTU-treated rats, there was a decrease in the level of OPA-1 (PTU effect: F_1,17_ = 10.03, *p* < 0.05), but neither VEN nor L-T4 separately administered evoked any impact on this change, whereas in co-treatment, no difference vs. WKY was observed (Figure 9B).

In the frontal cortex, no changes in the levels of the mitochondrial fission marker DNM1L were shown (Figure 9C); however, in the hippocampus, PTU administration resulted in the DNM1L downregulation (PTU effect: F_1,16_ = 7.26, *p* < 0.05), and applied therapy did not reverse the induced effect in this brain structure (Figure 9D).

### 3.11. Levels of Glucocorticoid Receptor (GR) and FKBP51-Factor Regulating GR-Function in the Brain

**Figure 10:**
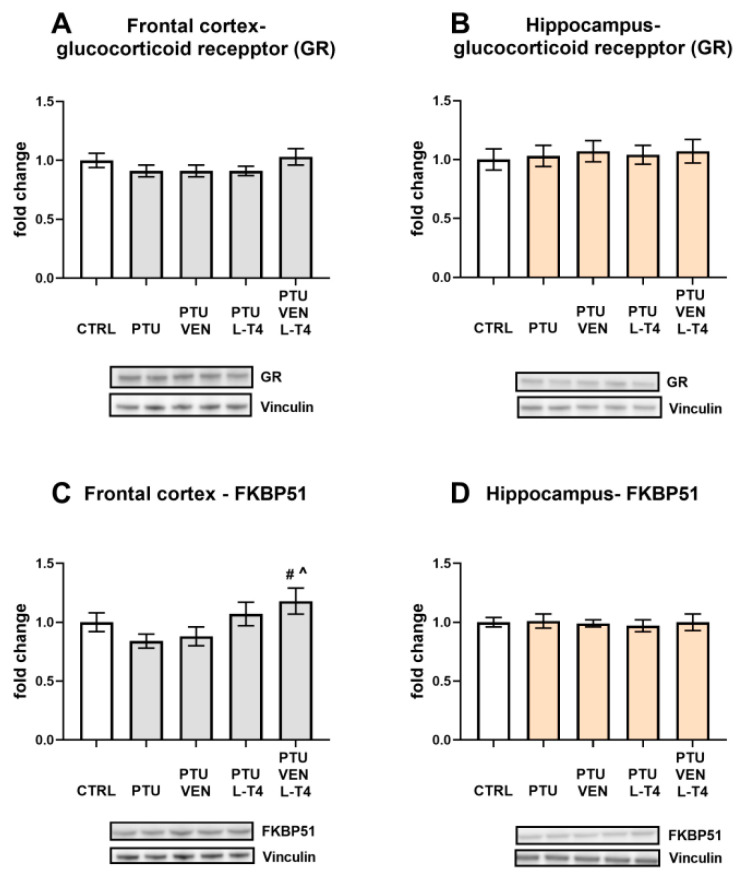
Effects of propylthiouracil (PTU) in Wistar Kyoto (WKY) rats and venlafaxine (VEN), levothyroxine (L-T4), and VEN + L-T4 administration on glucocorticoid receptor (GR) and FKBP51 in tissue homogenates of the frontal cortex (**A**,**C**) and the hippocampus (**B**,**D**) in PTU-treated WKY rats. The bands from the left: WKY, WKY + PTU, WKY + PTU + VEN, WKY + PTU + L-T4, and WKY + PTU + VEN + L-T4. The results are expressed as the mean ± SEM. # *p* < 0.05 vs. the WKY + PTU (coexisting depression and hypothyroidism group); ^ vs. the WKU + PTU + VEN group. *n* = 8. Statistics: one-way/factorial analysis of variance (ANOVA), post hoc comparison: Duncan test, when appropriate (See Supplementary Materials).

The level of GR was not changed by either PTU or the drugs in either brain structure (Figure 10A,B).

An analysis of the frontal cortex homogenates revealed an increased concentration of FKBP51 in rats receiving PTU together with combined treatment (VEN + L-T4) in comparison to the PTU group and to PTU animals that were administered VEN alone. An increased level of FKBP51 in this group was induced by L-T4 (L-T4 effect: F_1,28_ = 8.83, *p* < 0.05) (Figure 10C). In the hippocampus, no changes between the groups were observed (Figure 10D).

### 3.12. Levels of the Cell Damage Markers Caspase-1 p20, Bax, and Beclin1 in the Brain

**Figure 11:**
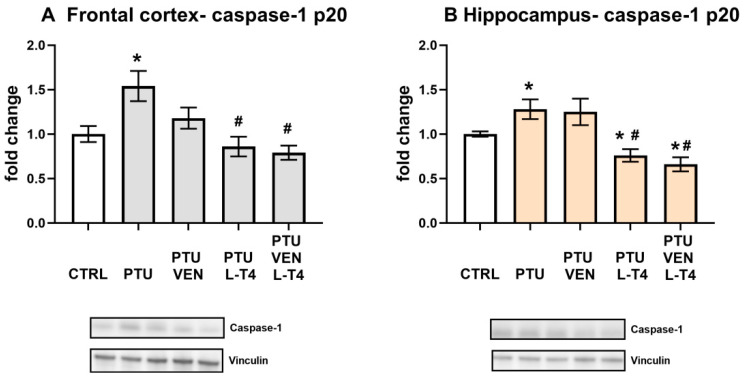
Effects of propylthiouracil (PTU) in Wistar Kyoto (WKY) rats and venlafaxine (VEN), levothyroxine (L-T4), and VEN + L-T4 administration on caspase-1 in tissue homogenates of the frontal cortex (**A**) and the hippocampus (**B**) in PTU-treated WKY rats. The bands from the left: WKY, WKY + PTU, WKY + PTU + VEN, WKY + PTU + L-T4, and WKY + PTU + VEN + L-T4. The results are expressed as the mean ± SEM. * *p* < 0.05 vs. the WKY (control group); # *p* < 0.05 vs. the WKY + PTU (coexisting depression and hypothyroidism group). *n* = 6–8. Statistics: one-way/factorial analysis of variance (ANOVA), post hoc comparison: Duncan test, when appropriate (See Supplementary Materials).

In both tested brain structures, PTU induced elevations in caspase-1 p20 levels (PTU effect: F_1,12_ = 7.64 in FCx; F_1,13_ = 5.72 in Hp, *p* < 0.05, Figure 11A,B). In the frontal cortex and hippocampus, this effect was inhibited by L-T4 in both treated groups, L-T4 alone, and L-T4 combined with VEN (L-T4 effect: F_1,26_ = 20.60 FCx; F_1,24_ = 23.65 in Hp, *p* < 0.05) (Figure 11A,B).

No changes in Bax and beclin 1 between the examined groups were detected in either the frontal cortex or the hippocampus (Table 2).

### 3.13. Concentrations of the Glutamate Transporters GLT-1 and GLAST in the Brain

**Figure 12:**
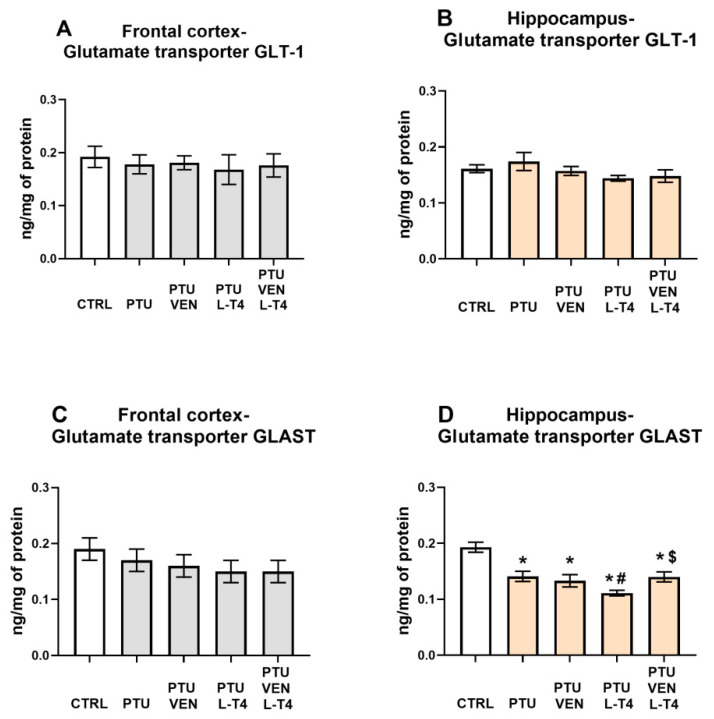
Effects of propylthiouracil (PTU) in Wistar Kyoto (WKY) rats and venlafaxine (VEN), levothyroxine (L-T4), and VEN + L-T4 administration on glutamate transporters GLT1 and GLAST in tissue homogenates of the frontal cortex (**A**,**C**) and the hippocampus (**B**,**D**) in PTU-treated WKY rats. The results are expressed as the mean ± SEM. * *p* < 0.05 vs. the WKY (control group); # *p* < 0.05 vs. the WKY + PTU (coexisting depression and hypothyroidism group), $ *p* < 0.05 vs. WKY + PTU + L-T4 group. *n* = 9–10. Statistics: one-way/factorial analysis of variance (ANOVA), post hoc comparison: Duncan test, when appropriate.

In the frontal cortex, no changes in GLT-1 (Figure 12A) or GLAST between the examined groups were detected (Figure 12C).

Similarly, neither PTU nor VEN and/or L-T4 affected the level of GLT-1 in the hippocampus (Figure 12B). However, PTU administration resulted in a reduction in GLAST-1, which was intensified by L-T4 administered alone but not by co-treatment with these two compounds (VEN x L-T4 interaction: F_1,35_ = 4.82) (Figure 12D).

## 4. Discussion

In this study, a hypothyroidism model was verified by measuring the levels of free T3 and T4 forms as well as TSH in the blood. In animals treated with PTU, the levels of fT3 and fT4 decreased significantly, while the concentration of TSH increased, indicating severe hypothyroidism. Administration of venlafaxine alone did not affect the levels of either hormone, while in animals receiving L-T4 alone and L-T4 with venlafaxine, not only were the PTU-induced changes reversed, but fT3, fT4, and TSH levels also showed the appearance of hyperthyroidism. L-T4 was used at a dose higher than the one that restores euthymia because this hormone is effective in the treatment of depression in humans at supraphysiological doses [38]. In our previous studies, we have shown that administering PTU for three weeks to WKY rats lowers their body weight while not affecting short-term memory and spatial memory, as determined in novel object recognition and novel object location tests, respectively, probably because the discrimination/preference indexes in these tests are much lower in this strain of rats than in Wistar animals [23,24]. Similarly, PTU did not affect the anxiety behavior evaluated in the elevated plus maze test or depression-like behavior assayed in the forced swim test, perhaps because the parameters determined in these tests were already different at baseline in WKY rats compared to Wistar rats, i.e., showing pro-depressive and pro-anxiety behaviors in WKY individuals [23,24]. Therefore, in the present study, we determined anxiety behavior using a different methodology, a light-dark box test, in which the anxiety-inducing action of PTU was observed. The applied therapies, especially the administration of L-T4 together with venlafaxine, tended to decrease the anxiolytic effect of PTU, but due to large interindividual variability, these differences did not reach significance. The hypothyroidism model used was also verified by determining the impacts of PTU and applied treatment on blood glucose and lipid metabolism markers. According to the results obtained previously [23], the present results also showed that administration of PTU increased the levels of total cholesterol and its HDL and LDL fractions and simultaneously decreased the level of triglycerides. Of these parameters, only LDL levels were normalized by L-T4 and L-T4 administered together with venlafaxine, while venlafaxine treatment alone did not show such a beneficial effect. Additionally, in the case of HDL, a favorable impact in animals receiving L-T4 alone and with venlafaxine, i.e., an increase in this protective cholesterol fraction, was demonstrated. In contrast to the beneficial effect of L-T4 alone and combined with venlafaxine on LDL and HDL levels, these animals experienced an increase in glucose levels.

Our results indicate that in the applied animal model of coexisting depression with anxiety and hypothyroidism, glycolysis was reduced compared to that in the depression model itself. In the frontal cortex, it was evidenced by the decreased levels of pyruvate and lactate in the cytosolic fraction, i.e., both end products of the glycolysis process. What is important, the decreased cytosolic lactate level was normalized in a similar way by venlafaxine, L-T4, and by the co-treatment, while the decreased level of pyruvate was increased more strongly in the frontal cortex of animals receiving venlafaxine plus L-T4 than those receiving venlafaxine alone, which indicated that L-T4 is able to potentiate the effect of venlafaxine. In the frontal cortex, there was not only a decrease in the level of glycolysis products but also a decrease in the level of pyruvate dehydrogenase in the mitochondrial fraction, which suggests a decrease in the supply of substrates for the Krebs cycle and therefore possibly also the limitations of this cycle. As with pyruvate levels, decreased expression of pyruvate dehydrogenase, an enzyme that connects the glycolysis process with the Krebs cycle, was normalized in WKY rats treated with L-T4 and venlafaxine. However, in the case of this enzyme, administration of either venlafaxine or L-T4 alone did not affect its level at all, which indicated that the antidepressant used had an effect on this parameter only after thyroid hormone deficiency had been compensated. In the hippocampus, lower levels of lactate in the cytosolic fraction in PTU-treated WKY rats in comparison to animals of this strain without hypothyroidism support the idea of glycolysis reduction. Lactate levels were normalized by both the administration of venlafaxine and venlafaxine together with L-T4, while in the case of pyruvate dehydrogenase, the tested drugs did not have a significant effect. In both the frontal cortex and the hippocampus, lactate levels were much higher than pyruvate levels, which is consistent with the present data showing that as a result of glycolysis, which is performed mostly in astrocytes, lactate is released from these cells into the extracellular fluid and delivered to neurons [39]. Neurons, as brain cells, need a large amount of energy stored in ATP to maintain their function and mainly carry out the process of oxidative phosphorylation. Lactate shuttling from astrocytes to neurons [40,41,42] is an important process that ensures the proper production of ATP by neurons, and disturbance of this process, which plays an important role not only in the pathogenesis of neurodegenerative diseases but also in depression, may also be due to altered expression of lactate transporters. Since we did not show differences in the expression of monocarboxylate transporter 2 (MCT2), which is involved in the transport of lactate to neurons, or MCT4, which is responsible for the transport of lactate to astrocytes, in any of the study groups, the metabolic changes observed in the model of coexisting depression and hypothyroidism were not due to changes in lactate transport. Since lactate is not only an end-product of glycolysis but may also act as an intercellular signaling molecule via G-protein-coupled receptor 81 (GPR81), also known as hydroxycarboxylic acid receptor 1-HCAR1 [43,44], we measured the level of this receptor in the brain; however, no changes were detected, which suggested that the observed effects were mainly metabolic.

Contrary to the effective reversal of hypothyroidism-induced decreases in lactate and pyruvate levels and pyruvate dehydrogenase expression in the frontal cortex of WKY rats by venlafaxine and/or L-T4, these drugs did not have any effect on respiratory chain complexes. The administration of PTU significantly decreased the expression of complexes I, III, and IV, while all the applied therapies (venlafaxine and L-T4 separately and in combination) not only did not inhibit these changes, but even in the case of complexes I and IV, there was a tendency to intensify the decrease. The reduction in the level of mitochondrial complexes in the model of depression and hypothyroidism occurred only in the frontal cortex and not in the hippocampus, which is consistent with our previous data and indicates a greater sensitivity of the frontal cortex to thyroid hormone deficiencies. Thus, among the metabolic disturbances observed in the model of coexisting depression and hypothyroidism, changes in the process of glycolysis and the transition from glycolysis to the Krebs cycle were normalized under the influence of T4 and/or venlafaxine administration, while the applied therapy did not affect the reduction in the expression of mitochondrial respiratory chain enzymes in the frontal cortex. Since not only the expression of particular enzymes but also mitochondrial dynamics are important in mitochondrial metabolism, the effects of hypothyroidism on the markers of mitochondrial fission and fusion were determined [45]. The reduction in the concentration of OPA-1, a fusion marker, in the hippocampus of WKY rats receiving PTU, indicates that in the model of depression and hypothyroidism, the process of mitochondrial dynamics could also be disrupted; however, this change was not normalized by L-T4 and venlafaxine but was attenuated only in the animals receiving the combined T4 and venlafaxine treatment. However, the influence of PTU and the therapy used on the dynamics of mitochondria in the hippocampus is difficult to explain and its physiological significance is difficult to evaluate because the expression levels of not only OPA-1 but also the fission marker DNM1L were reduced.

A well-described change in depression is the excessive concentration or action of glucocorticoids [46,47]. Many studies have shown that enhanced activity of the hypothalamic–pituitary–adrenal (HPA) axis in depression can be evoked by a reduction in the expression of glucocorticoid receptors (GRs) in the hippocampus or frontal cortex and, as a consequence, a weakening of the inhibitory feedback mechanism [4,48,49]. It has also been shown that in depression, there may be changes in the factors regulating GR functions and, consequently, the impaired potency of glucocorticoids [50]. No changes in GR expression were observed in any of the groups tested in either the hippocampus or the frontal cortex or in the level of FKBP51 in the hippocampus. The level of FKBP51 did not change in the frontal cortex in the model of coexisting depression and hypothyroidism, but an interesting change is the increase in levels of this protein in animals receiving both venlafaxine and L-T4 but not in rats treated with venlafaxine or L-T4 alone. FKBP-51 is a co-chaperone of GR that decreases GR translocation from the cytoplasm to the nucleus and thereby inhibits GR function [51]. The weakening of GR function in depression is considered to be a beneficial change because it may limit the adverse effects of glucocorticoids on the morphology and function of nerve cells. However, venlafaxine exerts this beneficial effect in the model of depression and hypothyroidism comorbidity only in the presence of thyroid hormone replacement. It is worth noting that both thyroid hormones and some antidepressants have neuroprotective effects. This fact may be important in their antidepressant properties because disturbances in synaptic plasticity in the frontal cortex and hippocampus in depression are well recognized [52]. Due to the neuroprotective effects of thyroid hormones, we checked whether lowering their levels in WKY rats would cause changes in selected markers of neurodegenerative processes. It was shown that PTU did not change the level of the major pro-apoptotic protein (Bax) or the marker of the autophagy process (beclin-1); conversely, it strongly increased the level of caspase-1, the enzyme involved in the activation of pro-inflammatory cytokine release. We have previously shown that the level of caspase-1 in the hippocampus is higher in WKY rats than in their control strain (Wistar rats) and that only in WKY rats does administration of PTU increase the expression of this enzyme [24]. In the present study, we confirmed an increase in caspase-1 levels in the hippocampus of WKY rats with hypothyroidism and found the same effect in the frontal cortex. The important roles of caspase-1, which is activated mainly by the NLRP3 inflammasome, involve the activation of inflammatory cytokines and the induction or intensification of neuroinflammation. In addition to immunoactivation, it has been shown that this enzyme, by affecting the function of the AMPA receptor, weakens long-term memory [53]. Interestingly, as in the brain structures in the present model, it has been found that in the heart, hypothyroidism also increases the concentration of caspase-1 protein, which suggests that this action may be one of the important mechanisms of hypothyroidism-induced tissue damage [54]. Importantly, the effects of PTU on the levels of caspase-1 in both examined brain structures were normalized by L-T4 and the combined administration of L-T4 and venlafaxine, whereas administration of venlafaxine alone was not effective.

It has been demonstrated that one of the neuroprotective mechanisms of action of thyroid hormones is decreasing the extracellular glutamate concentration and, thus, its toxic effects by enhancing its uptake into astrocytes [55]. However, there was no significant reduction in GLT-1, the main astrocytic glutamate transporter, which is responsible for more than 90% of glutamate uptake activity, in any of the examined brain structures in animals receiving PTU. The expression level of the GLAST transporter did not change in the frontal cortex, while in the hippocampus, its expression decreased in animals receiving PTU, and none of the applied treatments normalized its level, a result that is difficult to explain. Administration of L-T4 alone even enhanced this change. However, given the much smaller contribution of GLAST than GLT-1 to glutamate uptake, it seems that lowering hippocampal GLAST should not lead to a significant increase in extracellular glutamate levels, at least not by decreasing the expression levels of these transporters.

In summary, the obtained data demonstrated that in the model of coexisting depression and hypothyroidism, some metabolic changes, such as a reduction in the level of pyruvate dehydrogenase in the frontal cortex, were normalized only by the combined use of L-T4 with venlafaxine, and such treatment also inhibited changes in the pyruvate level in this brain structure more strongly than using L-T4 or venlafaxine alone. Contrary to the beneficial effects of L-T4 and/or venlafaxine on glycolysis and the transition from glycolysis to the Krebs cycle, neither of these drugs improved the changes in the expression of mitochondrial respiratory chain enzymes in the frontal cortex in WKY rats with hypothyroidism. An unfavorable change caused by the lack of thyroid hormones in WKY rats was the increase in the active form of caspase-1 in both examined brain structures, which indicated the possibility of intensifying proinflammatory processes, and this change was not normalized by venlafaxine alone without improvement of thyroid hormone deficiency. It should be noted, however, that the WKY rats used in this study as a model of depression, apart from depression-like behavior, also exhibit anxiety-like behavior, often observed in depression, and administration of PTU intensifies the anxiety-like behavior, therefore, it cannot be unequivocally determined whether the metabolic changes in the brain observed in the WKY + PTU model are more related to depression or anxiety.

Our results indicate that the use of thyroxine as an adjunctive therapy for depression may normalize neurotransmission not only by acting on neurons, as other authors suggest [13,25], but also by influencing the function of astrocytes and microglial cells, weakening disturbances in the glycolysis process and possibly also by reducing expression of the active form of caspase-1 to regulate the process of neuroinflammation, but this potential effect requires further research.

## 5. Conclusions

In recent years, studies on depression and anxiety disorders are increasingly focusing on defining the role of glial cells and metabolic disturbances in the brain in the pathogenesis of these diseases. The influence of thyroid hormones on biochemical processes in the brain affected in these diseases is only beginning to be studied, and getting to know them could further help to advance the diagnostic, as well as the therapy of these diseases. Our results indicate that in the treatment of patients with depression or anxiety disorders, it is important to determine the level of thyroid hormones and in the case of hypothyroidism, simultaneous administration of L-T4 with an antidepressant. An important issue is also the selection of an appropriate antidepressant in the treatment of depression with hypothyroidism, because as our study showed, although L-T4 enhances many of the effects of venlafaxine, none of these drugs normalized the reduction of the expression of the respiratory chain complexes in the frontal cortex, so this issue requires further study.

## Figures and Tables

**Table 1:** Effects of propylthiouracil (PTU) in Wistar Kyoto (WKY) rats and venlafaxine (VEN), levothyroxine (L-T4), VEN + L-T4 administration on hydroxycarboxylic acid receptor 1: HCAR1 and monocarboxylate transporters: MCT2 and MCT4 in tissue homogenates of the frontal cortex and the hippocampus in PTU-treated WKY rats. The bands from the left: WKY, WKY + PTU, WKY + PTU + VEN, WKY + PTU + L-T4, and WKY + PTU + VEN + L-T4. The results are expressed as the mean ± SEM. *n* = 6–8. Statistics: one-way/factorial analysis of variance (ANOVA) (See Supplementary Materials).

Protein	Brain Structure	WKY	WKY + PTU	WKY + PTU + VEN	WKY + PTU + L-T4	WKY + PTU + VEN + L-T4
**HCAR1**	Frontal Cortex	1.00 ± 0.10	0.94 ± 0.07	0.97 ± 0.11	1.01 ± 0.11	0.93 ± 0.08
	Hippocampus	1.00 ± 0.08	0.92 ± 0.08	0.92 ± 0.09	0.98 ± 0.09	1.00 ± 0.11
**MCT2**	Frontal Cortex	1.00 ± 0.11	1.08 ± 0.13	0.95 ± 0.07	1.10 ± 0.09	1.00 ± 0.08
	Hippocampus	1.00 ± 0.08	0.89 ± 0.06	0.90 ± 0.04	0.96 ± 0.07	0.85 ± 0.05
**MCT4**	Frontal Cortex	1.00 ± 0.07	0.80 ± 0.08	0.91 ± 0.07	1.02 ± 0.08	0.87 ± 0.08
	Hippocampus	1.00 ± 0.08	0.82 ± 0.08	0.86 ± 0.06	0.89 ± 0.08	0.84 ± 0.04
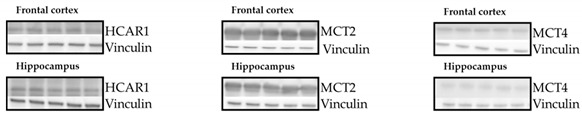						

**Table 2:** Effects of propylthiouracil (PTU) in Wistar Kyoto (WKY) rats and venlafaxine (VEN), levothyroxine (L-T4), and VEN + L-T4 administration on Bax and beclin-1 proteins in tissue homogenates of the frontal cortex and hippocampus in PTU-treated WKY rats. The bands from the left: WKY, WKY + PTU, WKY + PTU + VEN, WKY + PTU + L-T4, and WKY PTU VEN L-T4. The results are expressed as the mean ± SEM. *n* = 7–8. Statistics: one-way/factorial analysis of variance (ANOVA) (See Supplementary Materials).

Protein	Brain Structure	WKY	WKY + PTU	WKY + PTU + VEN	WKY + PTU + L-T4	WKY + PTU + VEN + L-T4
**Bax**	Frontal Cortex	1.00 ± 0.08	0.94 ± 0.07	0.92 ± 0.05	0.99 ± 0.06	0.98 ± 0.07
	Hippocampus	1.00 ± 0.06	1.01 ± 0.05	1.01 ± 0.07	1.00 ± 0.06	1.02 ± 0.06
**beclin 1**	Frontal Cortex	1.00 ± 0.07	0.91 ± 0.07	0.88 ± 0.08	0.87 ± 0.07	0.88 ± 0.07
	Hippocampus	1.00 ± 0.07	0.91 ± 0.04	0.92 ± 0.06	0.94 ± 0.06	0.88 ± 0.05
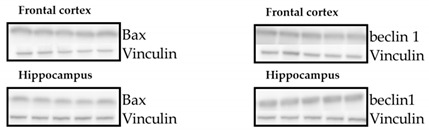						

## Data Availability

The data that support the findings of this study are available from the corresponding author upon reasonable request from qualified researchers.

## Supplementary Materials

The following are available online at https://www.mdpi.com/article/10.3390/cells10061394/s1.
