# Supplementary material for: Venlafaxine and L-Thyroxine Treatment Combination: Impact on Metabolic and Synaptic Plasticity Changes in an Animal Model of Coexisting Depression and Hypothyroidism
Source: Cells. 2021 Jun 5;10(6):1394. doi: 10.3390/cells10061394 (PMC8227539; doi:10.3390/cells10061394)
Supplement: Supplementary file 1 [file cells-10-01394-s001.zip › cells-1217325-supplementary.pdf]

OXPHOS Frontal cortex, membrane 1

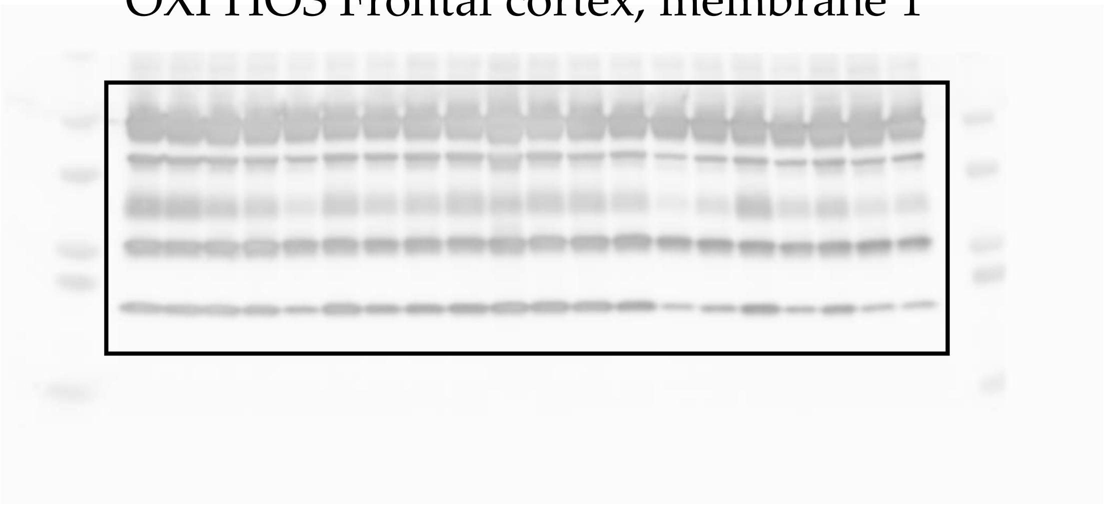

OXPHOS Frontal cortex, membrane 2

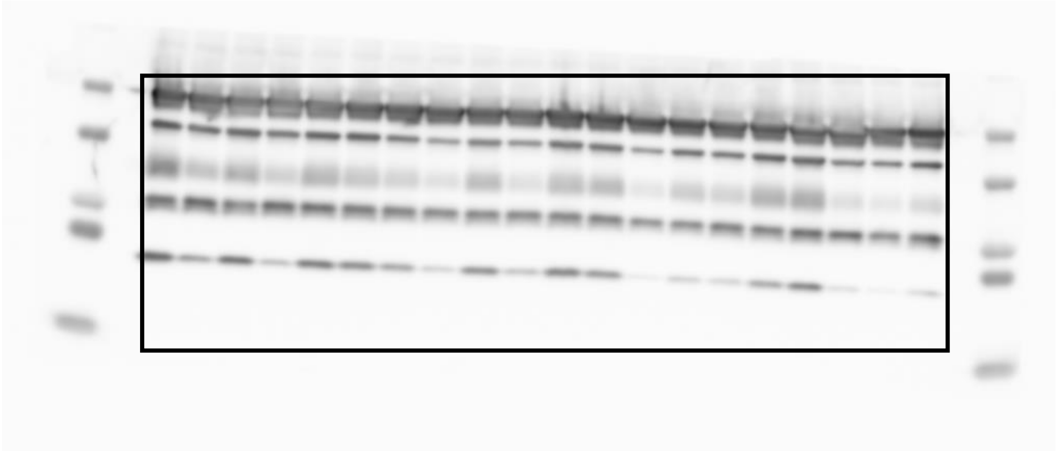

Vinculin as loading control to OXPHOS Frontal cortex, membrane 1 and 2

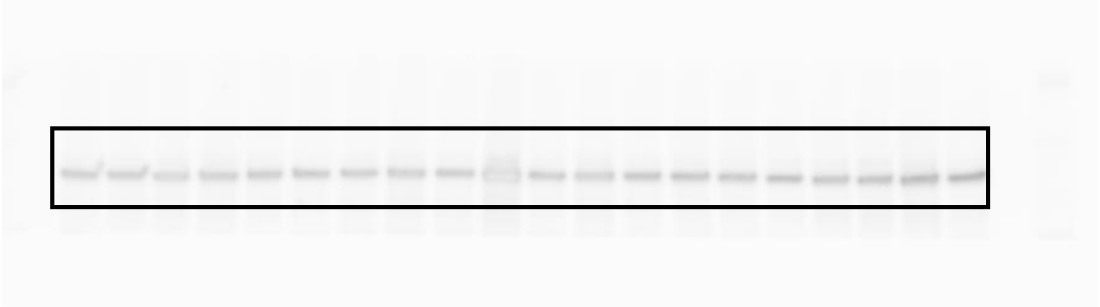

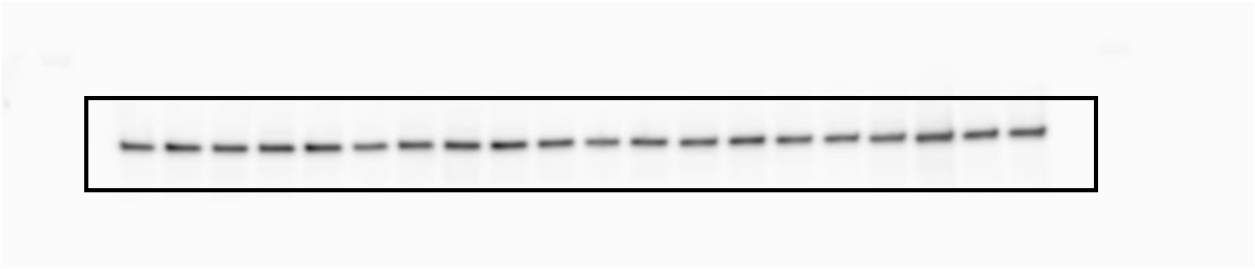

The bands from the left: WKY, WKY+PTU,WKY+PTU+VEN, WKY+PTU+T4, WKY+PTU+T4+VEN, WKY, WKY+PTU,WKY+PTU+VEN, WKY+PTU+T4, WKY+PTU+T4+VEN, WKY, WKY+PTU,WKY+PTU+VEN, WKY+PTU+T4, WKY+PTU+T4+VEN, WKY, WKY+PTU,WKY+PTU+VEN, WKY+PTU+T4, WKY+PTU+T4+VEN

The results of the experiment are shown in the Figure 8

OXPHOS Hippocampus, membrane 1

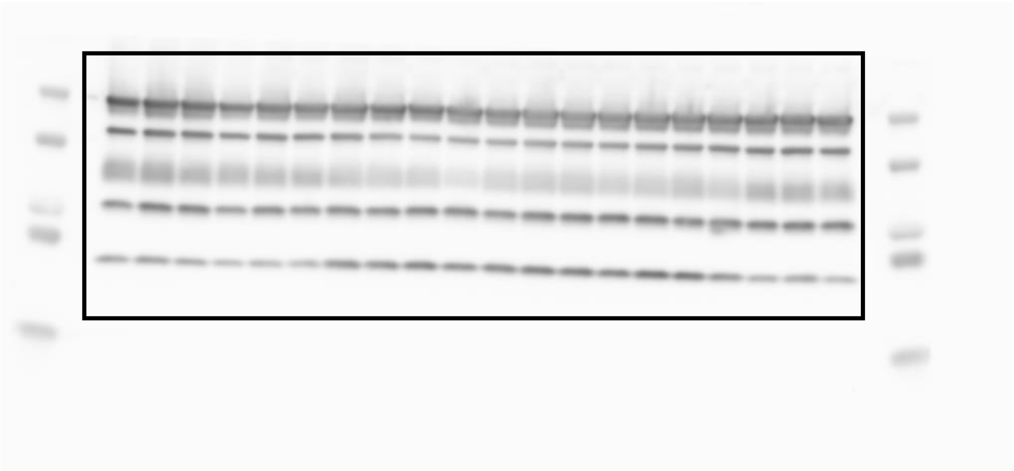

Vinculin as loading control to OXPHOS Hippocampus, membrane 1 and 2

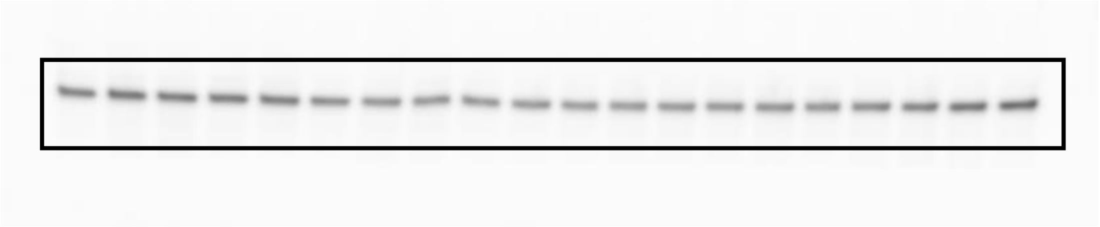

OXPHOS Hippocampus, membrane 2

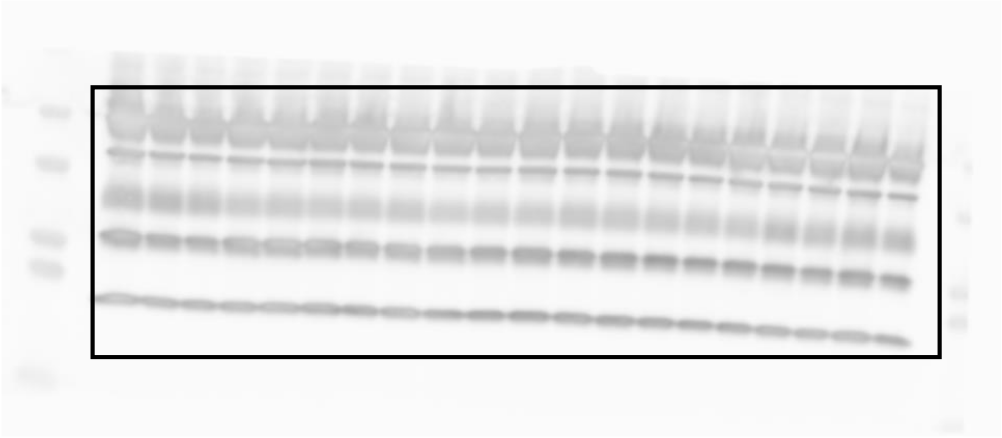

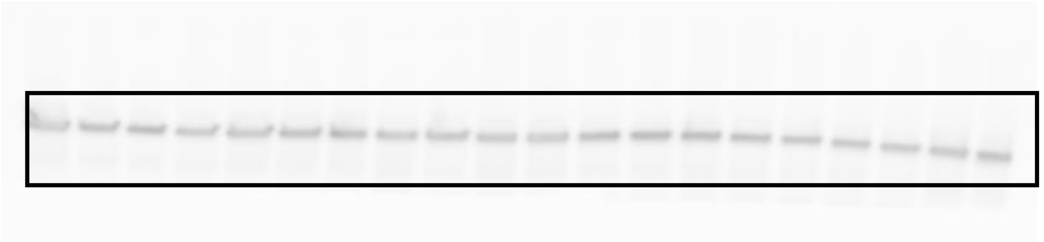

The bands from the left: WKY, WKY+PTU,WKY+PTU+VEN, WKY+PTU+T4, WKY+PTU+T4+VEN, WKY, WKY+PTU,WKY+PTU+VEN, WKY+PTU+T4, WKY+PTU+T4+VEN, WKY, WKY+PTU,WKY+PTU+VEN, WKY+PTU+T4, WKY+PTU+T4+VEN, WKY, WKY+PTU,WKY+PTU+VEN, WKY+PTU+T4, WKY+PTU+T4+VEN

The results of the experiment are shown in the Figure 8

GR Frontal cortex, membrane 1

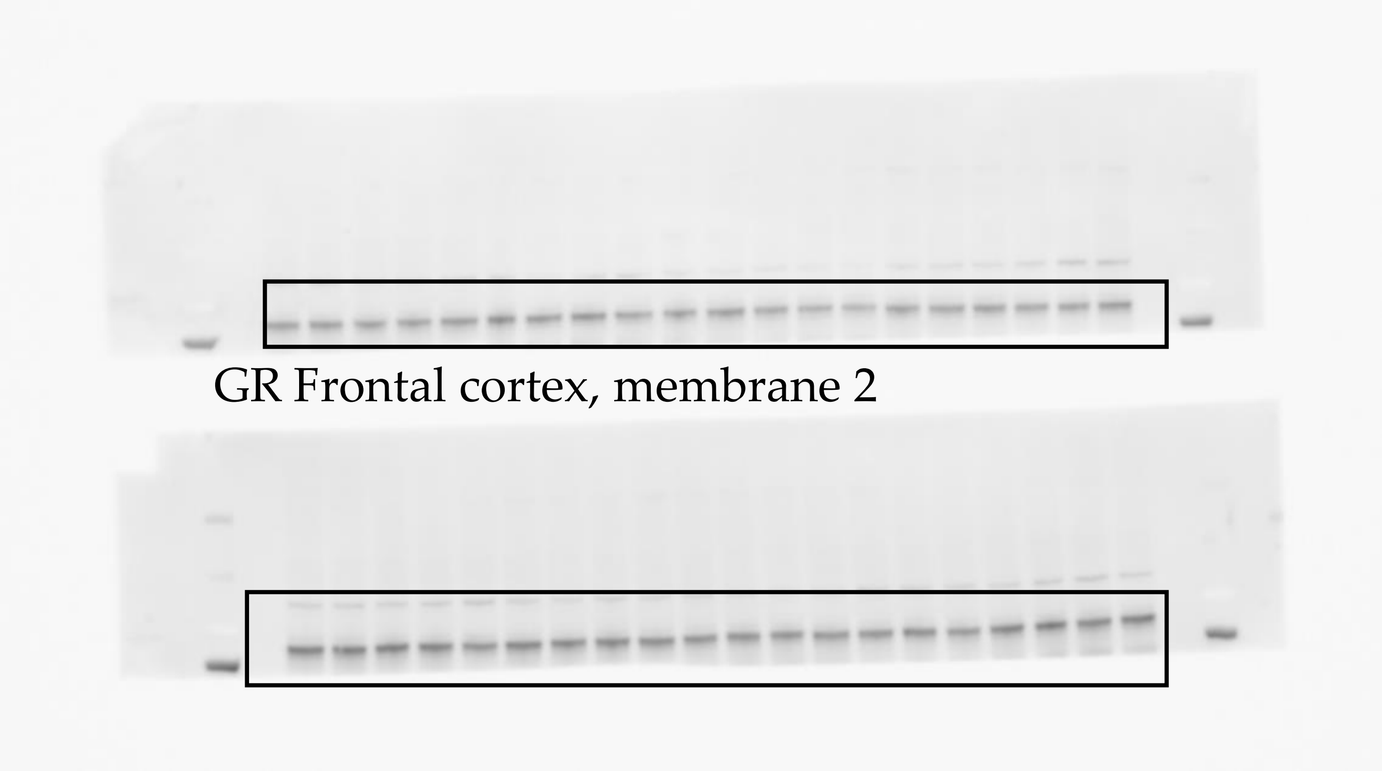

Vinculin as loading control to GR Frontal Cortex, membrane 1 and 2

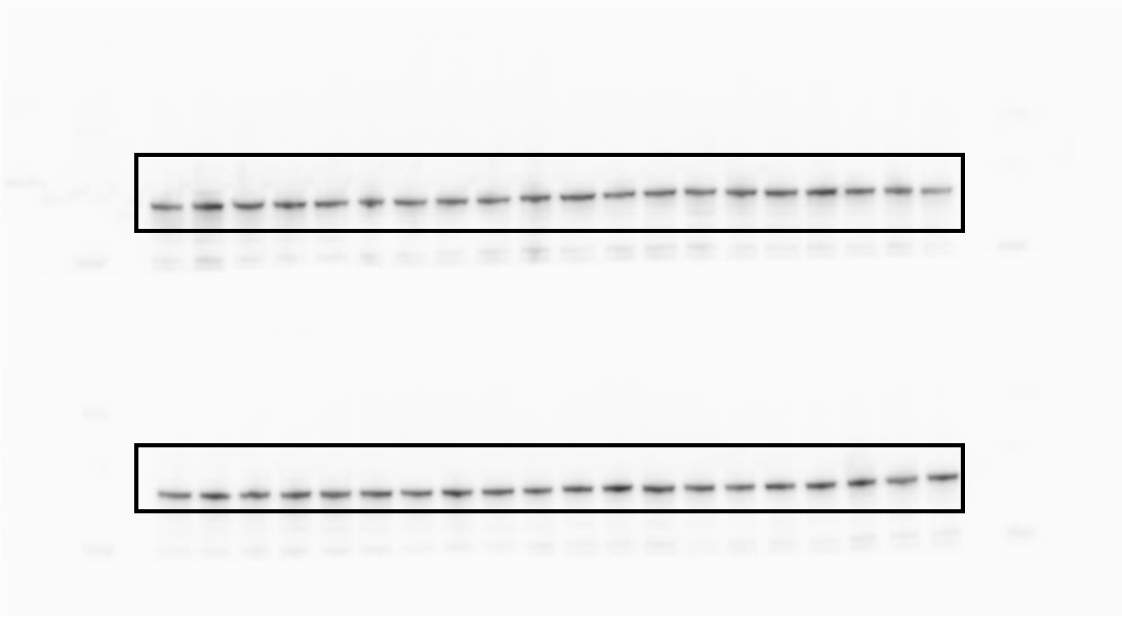

The bands from the left: WKY, WKY+PTU,WKY+PTU+VEN, WKY+PTU+T4, WKY+PTU+T4+VEN, WKY, WKY+PTU,WKY+PTU+VEN, WKY+PTU+T4, WKY+PTU+T4+VEN, WKY, WKY+PTU,WKY+PTU+VEN, WKY+PTU+T4, WKY+PTU+T4+VEN, WKY, WKY+PTU,WKY+PTU+VEN, WKY+PTU+T4, WKY+PTU+T4+VEN

The results of the experiment are shown in the Figure 10

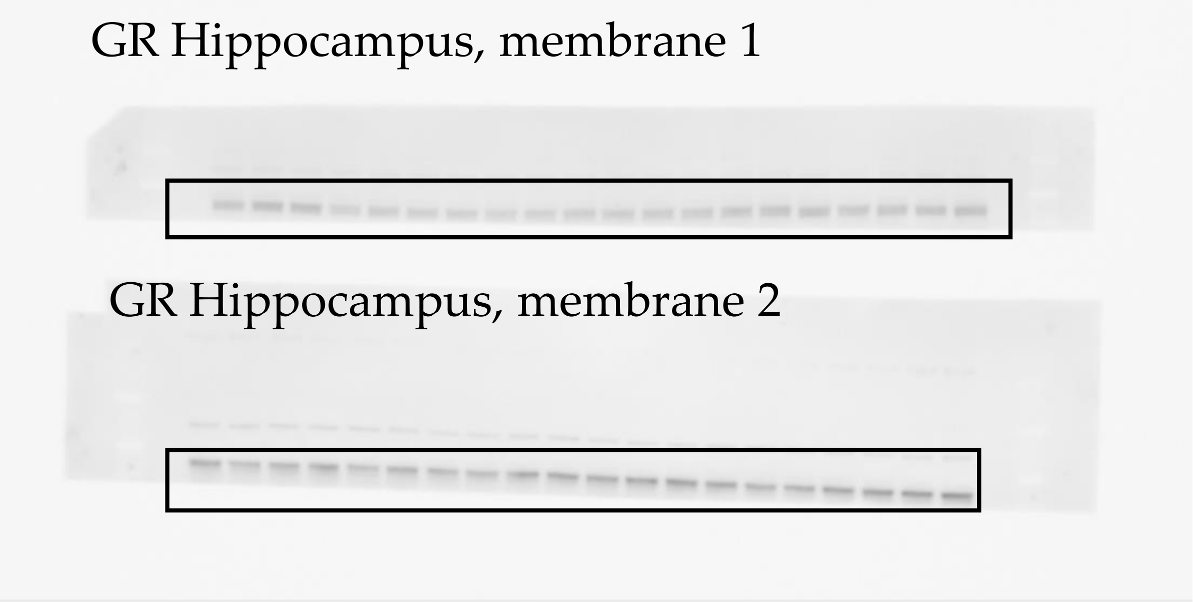

Vinculin as loading control to GR Hippocampus, membrane 1 and 2

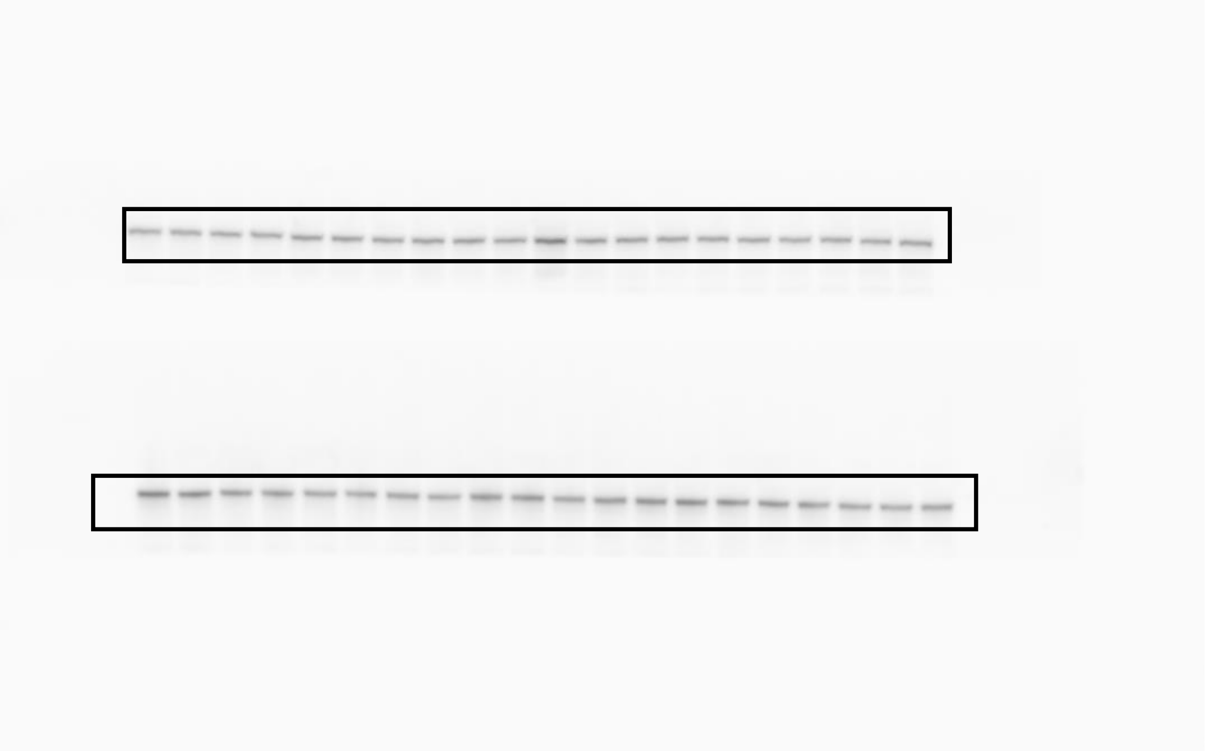

The bands from the left: WKY, WKY+PTU,WKY+PTU+VEN, WKY+PTU+T4, WKY+PTU+T4+VEN, WKY, WKY+PTU,WKY+PTU+VEN, WKY+PTU+T4, WKY+PTU+T4+VEN, WKY, WKY+PTU,WKY+PTU+VEN, WKY+PTU+T4, WKY+PTU+T4+VEN, WKY, WKY+PTU,WKY+PTU+VEN, WKY+PTU+T4, WKY+PTU+T4+VEN

The results of the experiment are shown in the Figure 10

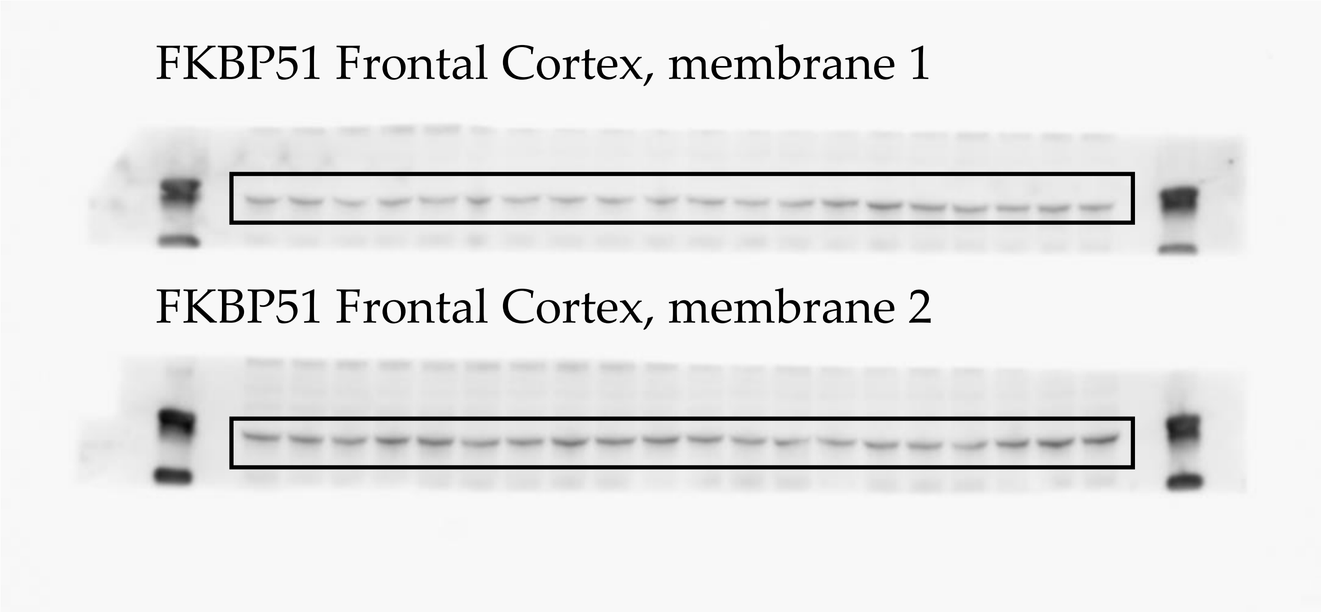

Vinculin as loading control to FKBP51 Frontal Cortex, membrane 1 and 2

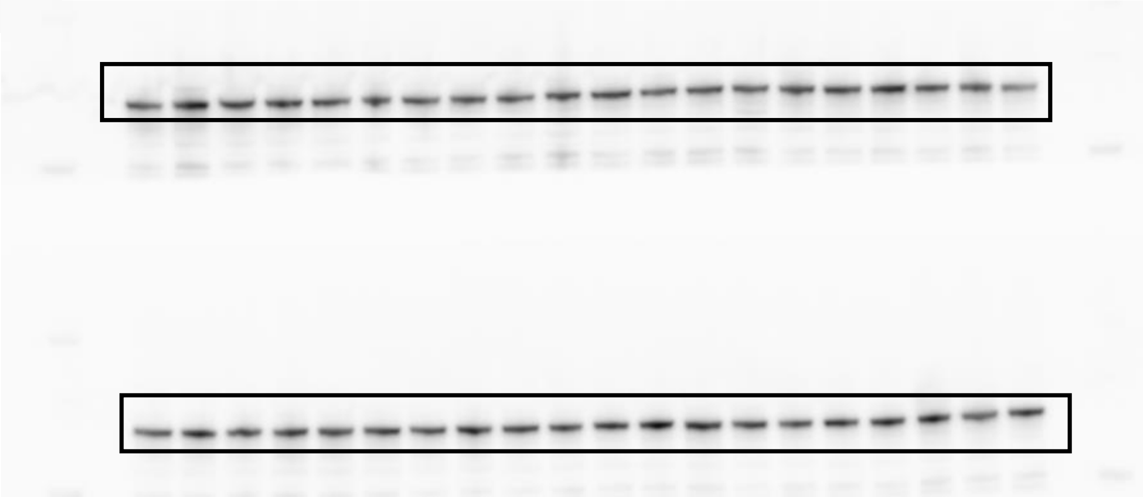

The bands from the left: WKY, WKY+PTU,WKY+PTU+VEN, WKY+PTU+T4, WKY+PTU+T4+VEN, WKY, WKY+PTU,WKY+PTU+VEN, WKY+PTU+T4, WKY+PTU+T4+VEN, WKY, WKY+PTU,WKY+PTU+VEN, WKY+PTU+T4, WKY+PTU+T4+VEN, WKY, WKY+PTU,WKY+PTU+VEN, WKY+PTU+T4, WKY+PTU+T4+VEN

The results of the experiment are shown in the Figure 10

FKBP51 Hippocampus, membrane 1

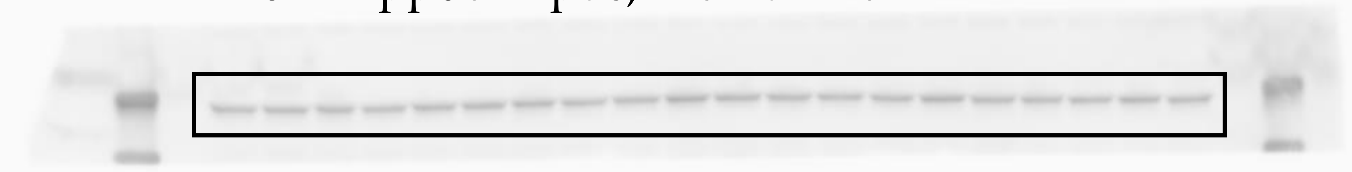

FKBP51 Hippocampus, membrane 2

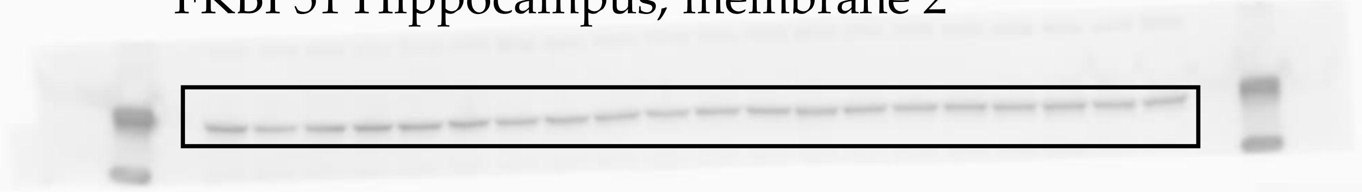

Vinculin as loading control to FKBP51 Hippocampus, membrane 1 and 2

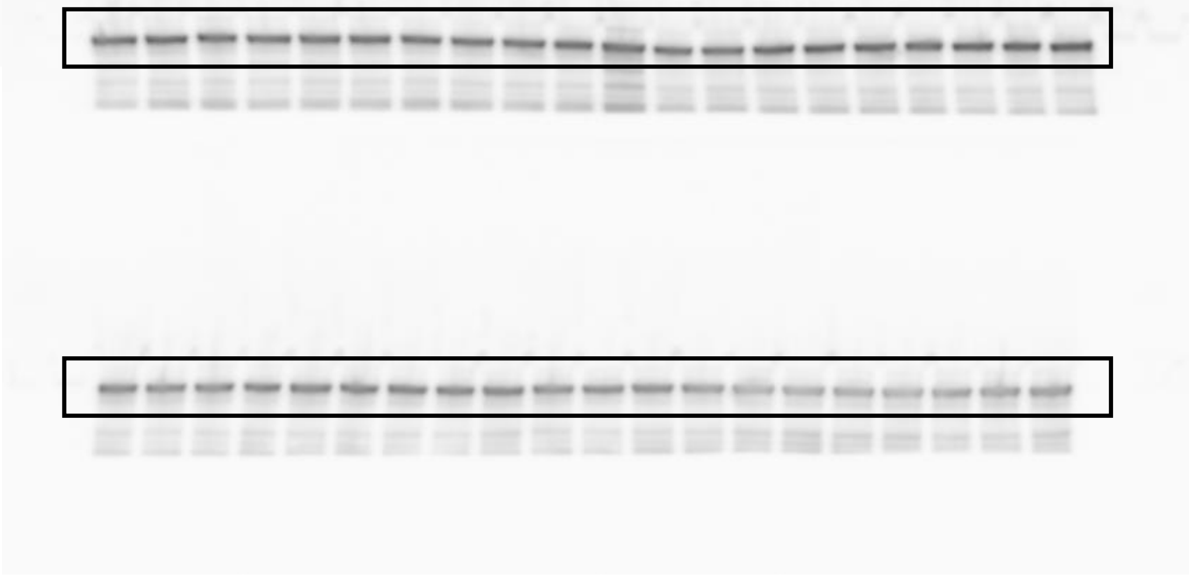

The bands from the left: WKY, WKY+PTU,WKY+PTU+VEN, WKY+PTU+T4, WKY+PTU+T4+VEN, WKY, WKY+PTU,WKY+PTU+VEN, WKY+PTU+T4, WKY+PTU+T4+VEN, WKY, WKY+PTU,WKY+PTU+VEN, WKY+PTU+T4, WKY+PTU+T4+VEN, WKY, WKY+PTU,WKY+PTU+VEN, WKY+PTU+T4, WKY+PTU+T4+VEN

The results of the experiment are shown in the Figure 10

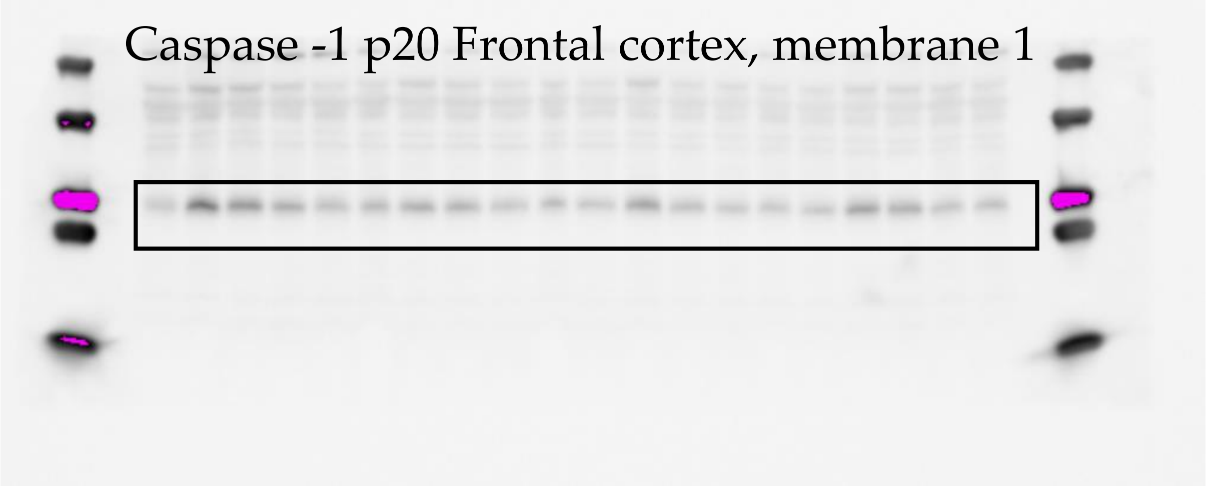

Vinculin as loading control to Caspase-1 p20
Frontal cortex, membrane 1 and 2

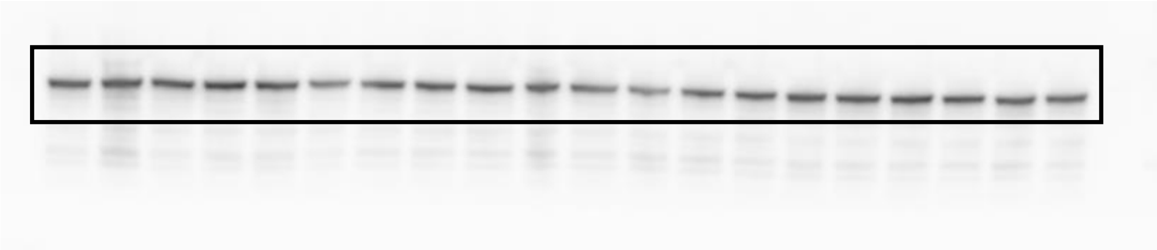

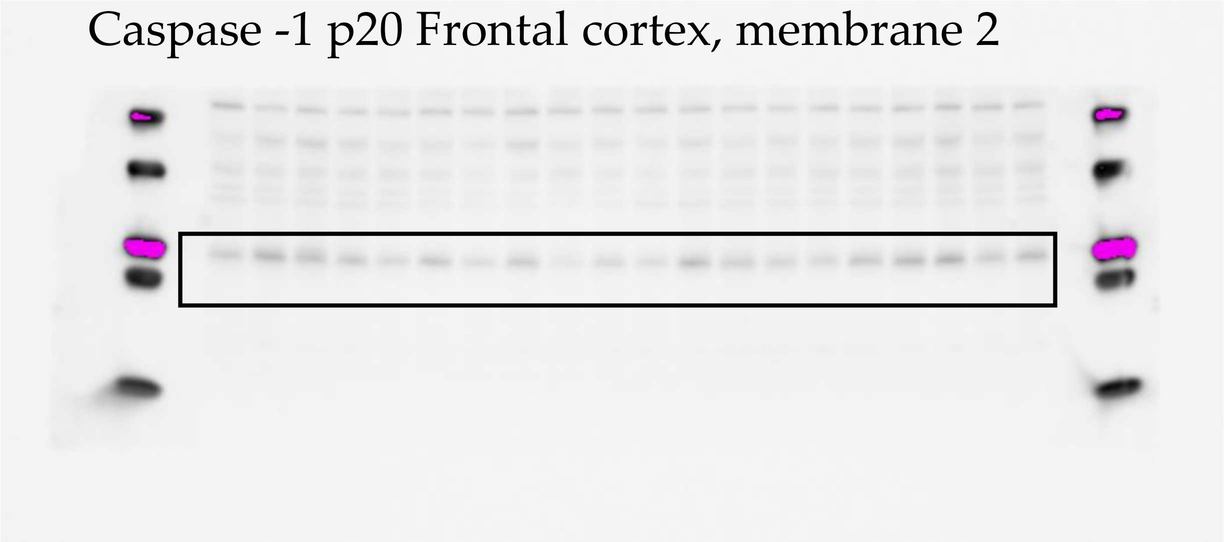

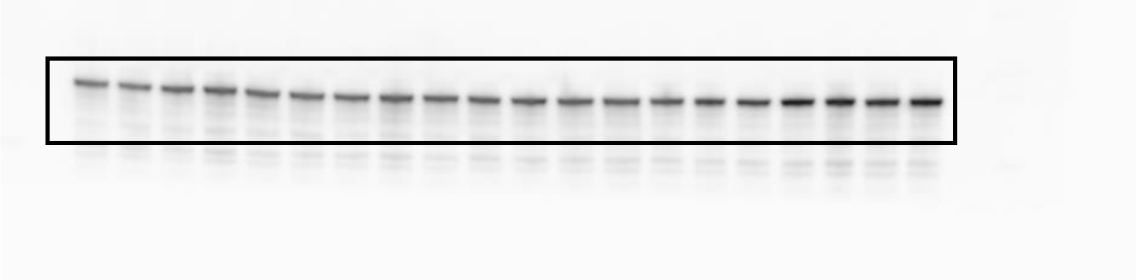

The bands from the left: WKY, WKY+PTU,WKY+PTU+VEN, WKY+PTU+T4, WKY+PTU+T4+VEN, WKY, WKY+PTU,WKY+PTU+VEN, WKY+PTU+T4, WKY+PTU+T4+VEN, WKY, WKY+PTU,WKY+PTU+VEN, WKY+PTU+T4, WKY+PTU+T4+VEN, WKY, WKY+PTU,WKY+PTU+VEN, WKY+PTU+T4, WKY+PTU+T4+VEN

The results of the experiment are shown in the Figure 11

Caspase -1 p20 Hippocampus, membrane 1

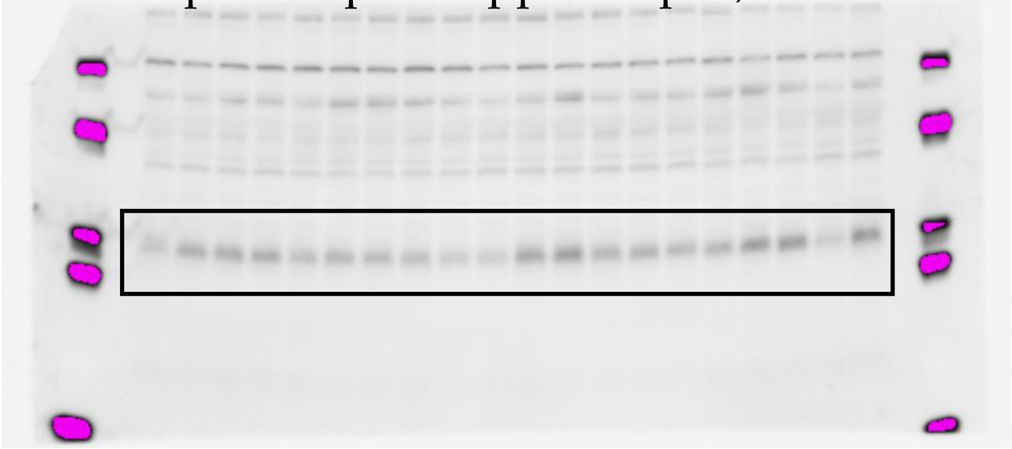

Vinculin as loading control to Caspase-1 p20 Hippocampus, membrane 1 and 2

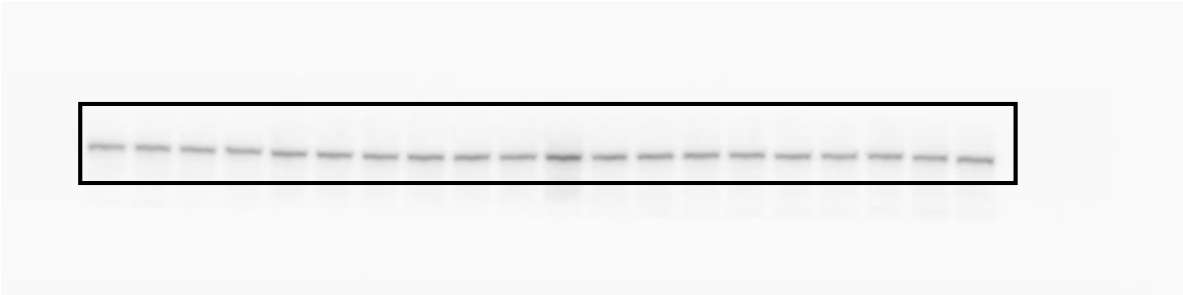

Caspase -1 p20 Hippocampus, membrane 2

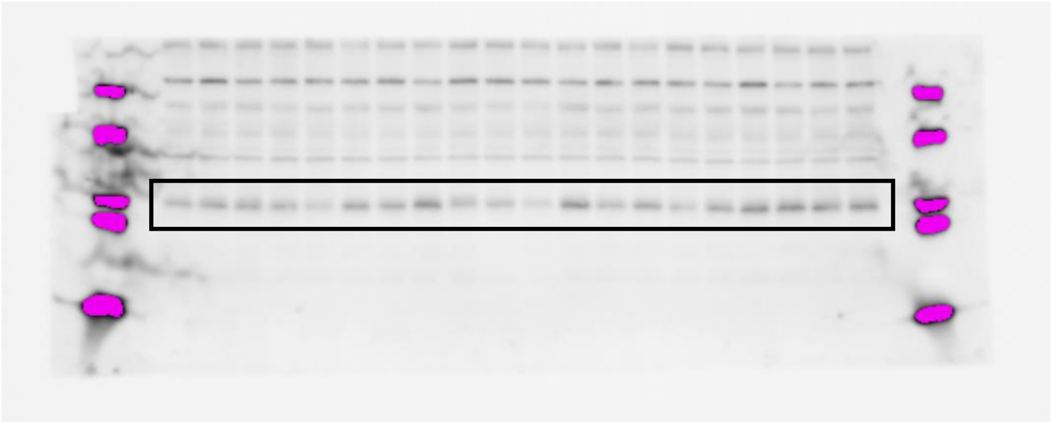

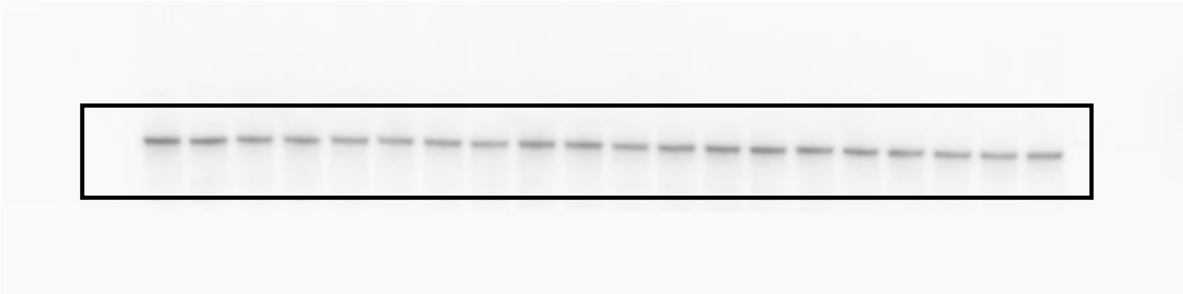

The bands from the left: WKY, WKY+PTU,WKY+PTU+VEN, WKY+PTU+T4, WKY+PTU+T4+VEN, WKY, WKY+PTU,WKY+PTU+VEN, WKY+PTU+T4, WKY+PTU+T4+VEN, WKY, WKY+PTU,WKY+PTU+VEN, WKY+PTU+T4, WKY+PTU+T4+VEN, WKY, WKY+PTU,WKY+PTU+VEN, WKY+PTU+T4, WKY+PTU+T4+VEN

The results of the experiment are shown in the Figure 11

MCT2 Frontal Cortex, membrane 1

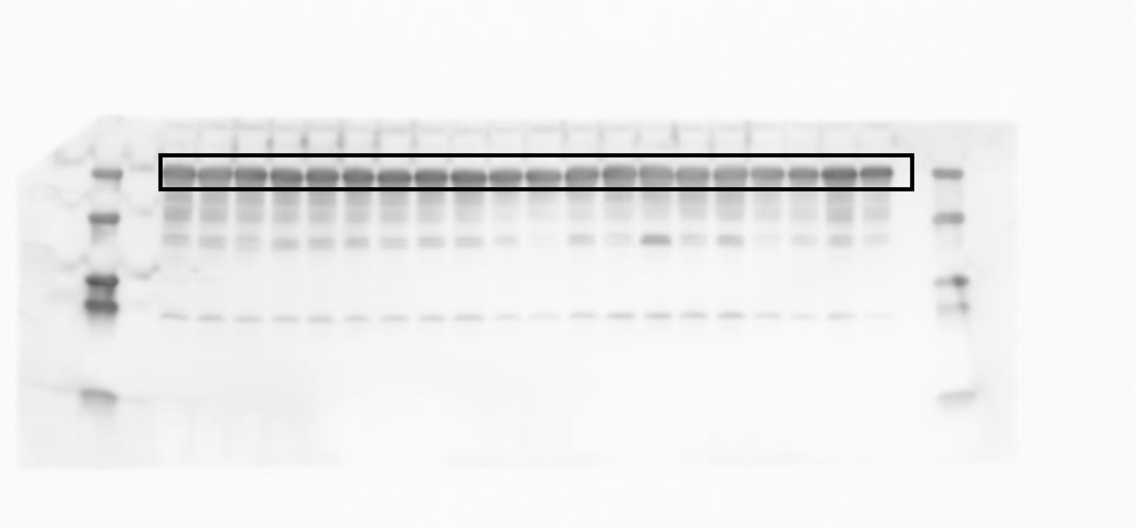

MCT2 Frontal Cortex, membrane 2

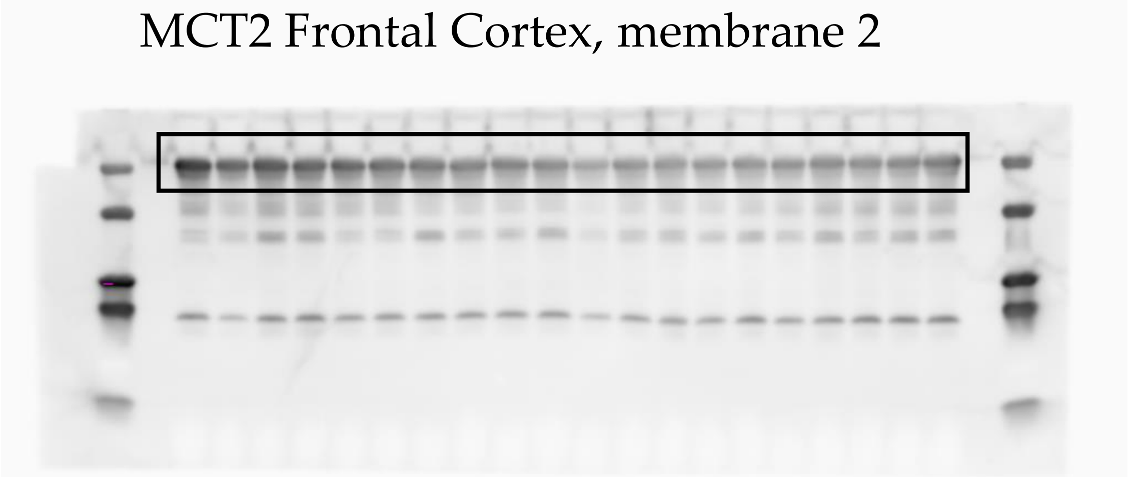

Vinculin as loading control to MCT2 Frontal Cortex, membrane 1 and 2

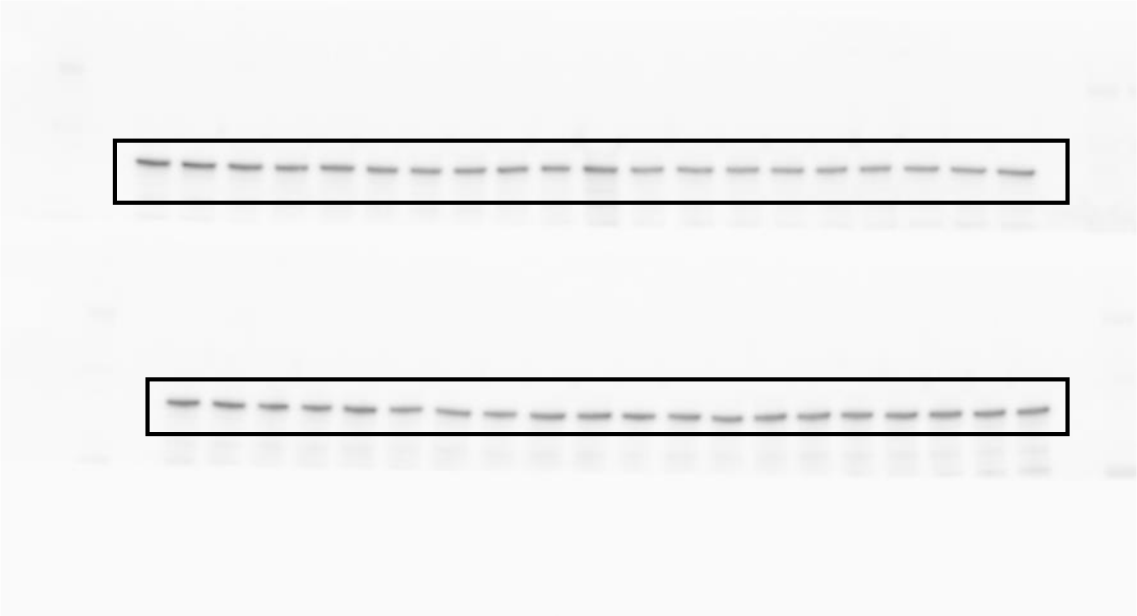

The bands from the left: WKY, WKY+PTU,WKY+PTU+VEN, WKY+PTU+T4, WKY+PTU+T4+VEN, WKY, WKY+PTU,WKY+PTU+VEN, WKY+PTU+T4, WKY+PTU+T4+VEN, WKY, WKY+PTU,WKY+PTU+VEN, WKY+PTU+T4, WKY+PTU+T4+VEN, WKY, WKY+PTU,WKY+PTU+VEN, WKY+PTU+T4, WKY+PTU+T4+VEN

The results of the experiment are shown in the Table 1

MCT2 Hippocampus, membrane 1

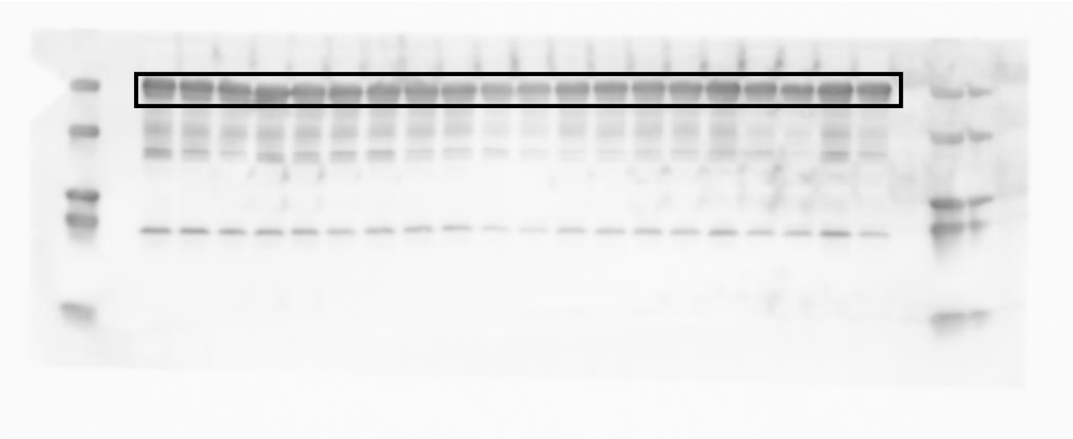

MCT2 Hippocampus, membrane 2

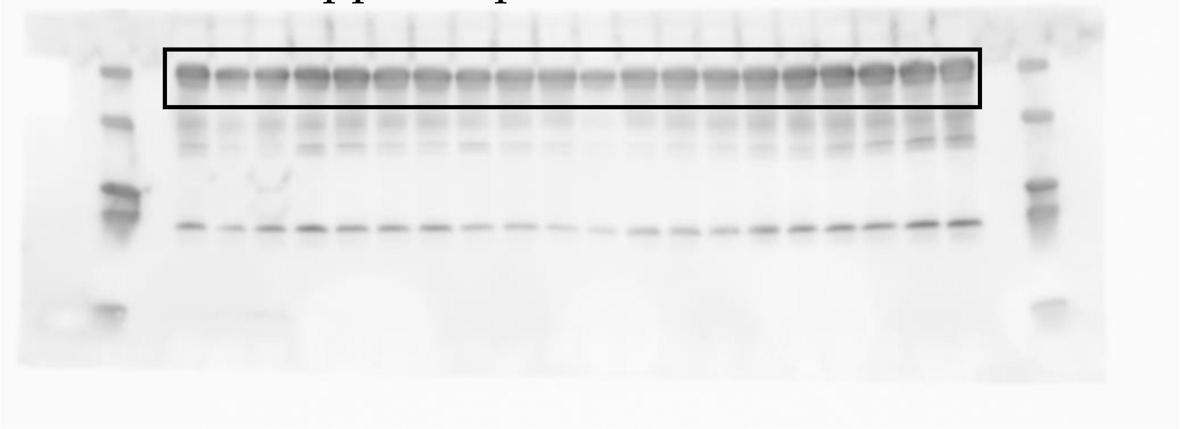

Vinculin as loading control to MCT2 Hippocampus, membrane 1 and 2

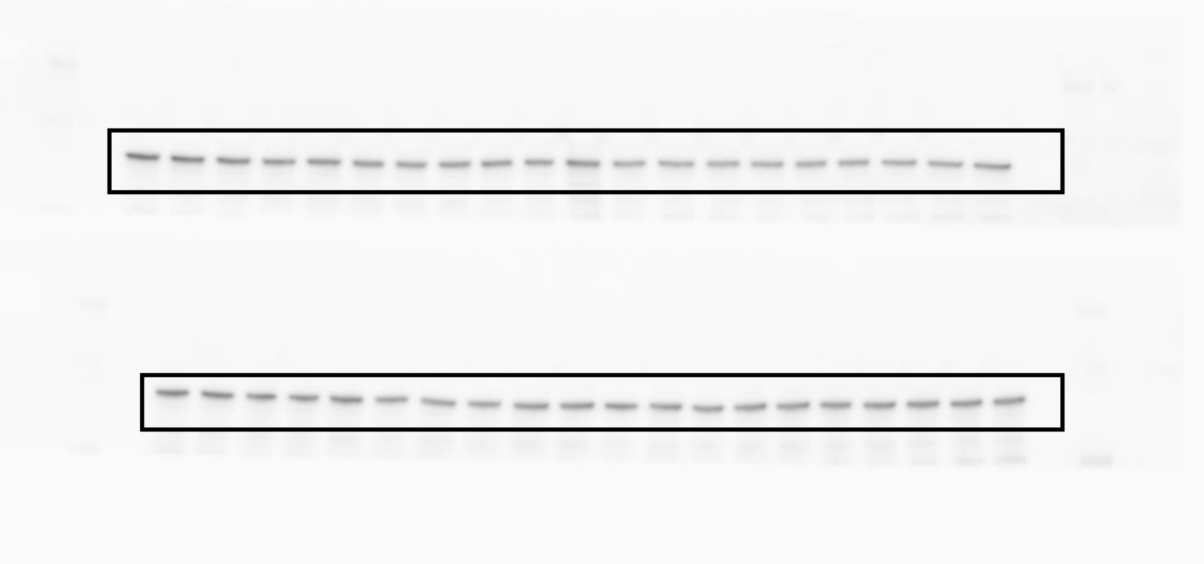

The bands from the left: WKY, WKY+PTU,WKY+PTU+VEN, WKY+PTU+T4, WKY+PTU+T4+VEN, WKY, WKY+PTU,WKY+PTU+VEN, WKY+PTU+T4, WKY+PTU+T4+VEN, WKY, WKY+PTU,WKY+PTU+VEN, WKY+PTU+T4, WKY+PTU+T4+VEN, WKY, WKY+PTU,WKY+PTU+VEN, WKY+PTU+T4, WKY+PTU+T4+VEN

The results of the experiment are shown in the Table 1

MCT4 Frontal Cortex, membrane 1

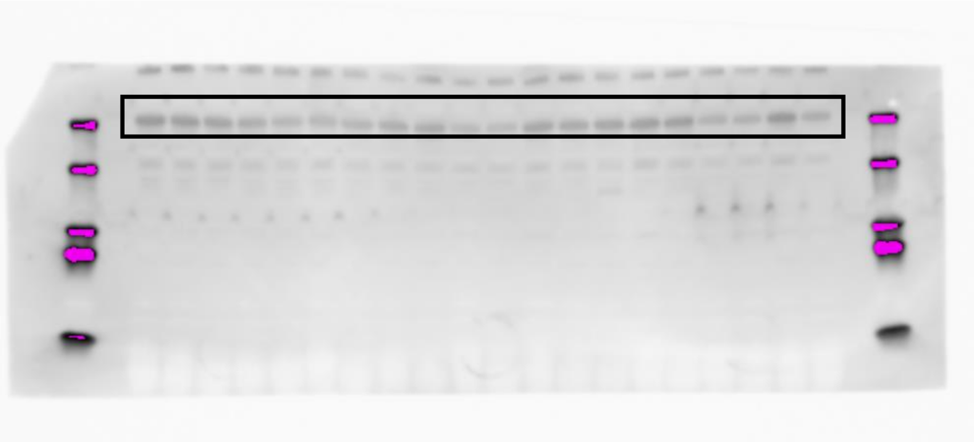

MCT4 Frontal Cortex, membrane 2

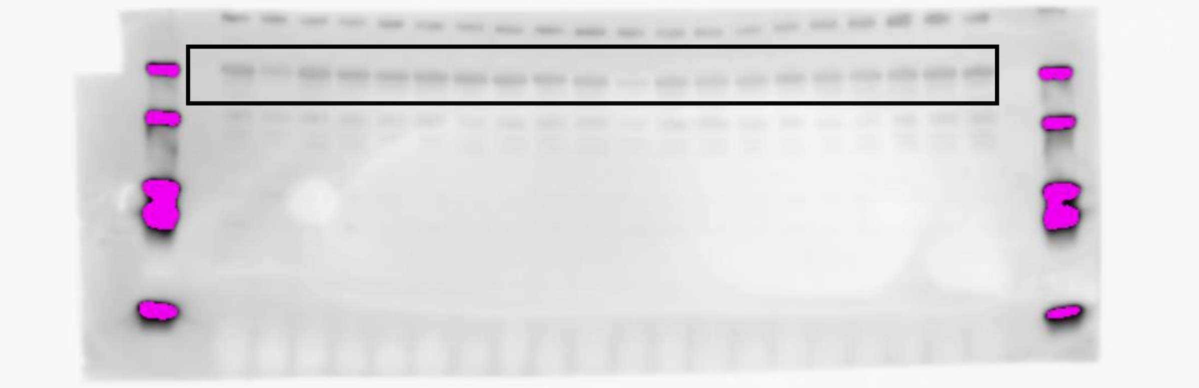

Vinculin as loading control to MCT4 Frontal Cortex, membrane 1 and 2

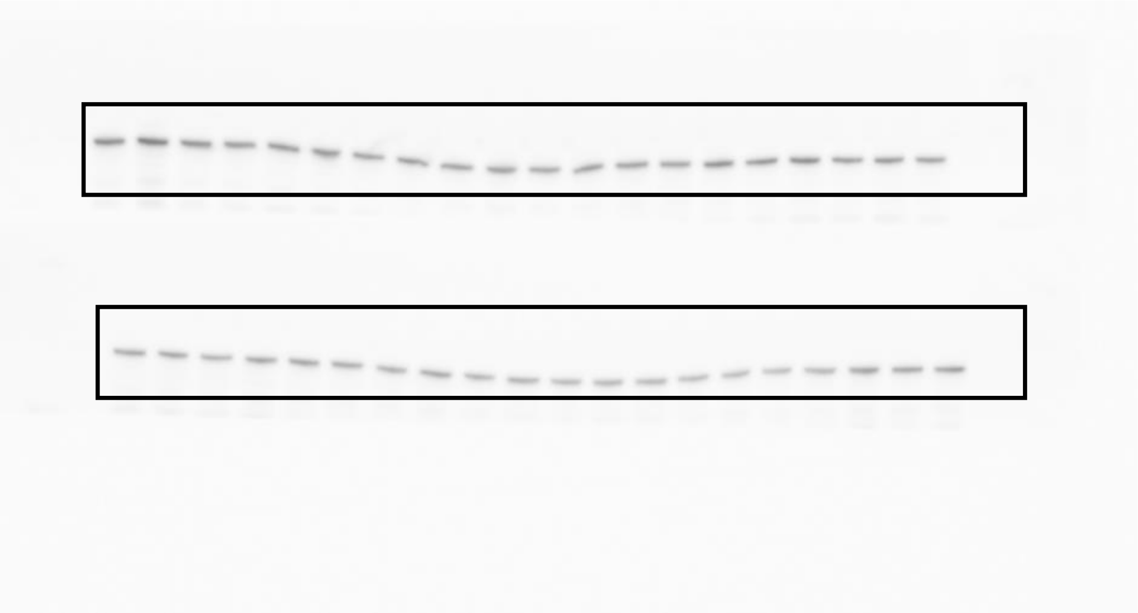

The bands from the left: WKY, WKY+PTU,WKY+PTU+VEN, WKY+PTU+T4, WKY+PTU+T4+VEN, WKY, WKY+PTU,WKY+PTU+VEN, WKY+PTU+T4, WKY+PTU+T4+VEN, WKY, WKY+PTU,WKY+PTU+VEN, WKY+PTU+T4, WKY+PTU+T4+VEN, WKY, WKY+PTU,WKY+PTU+VEN, WKY+PTU+T4, WKY+PTU+T4+VEN

The results of the experiment are shown in the Table 1

MCT4 Hippocampus, membrane 1

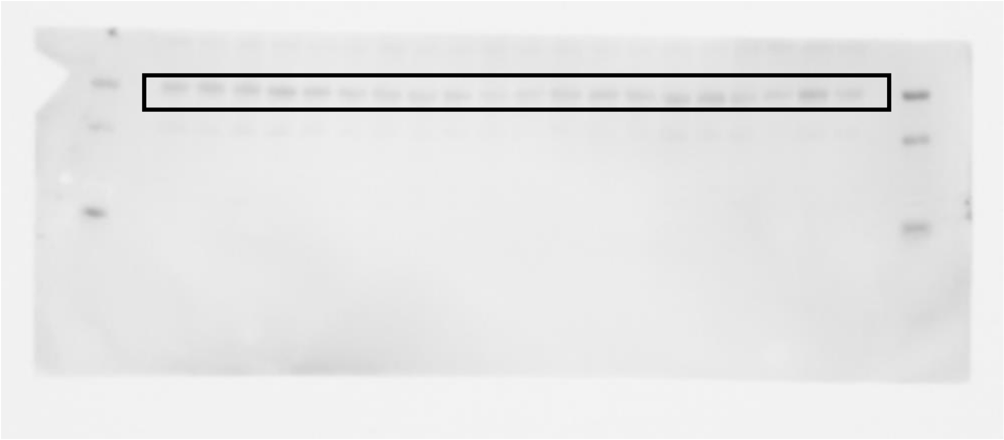

MCT4 Hippocampus, membrane 2

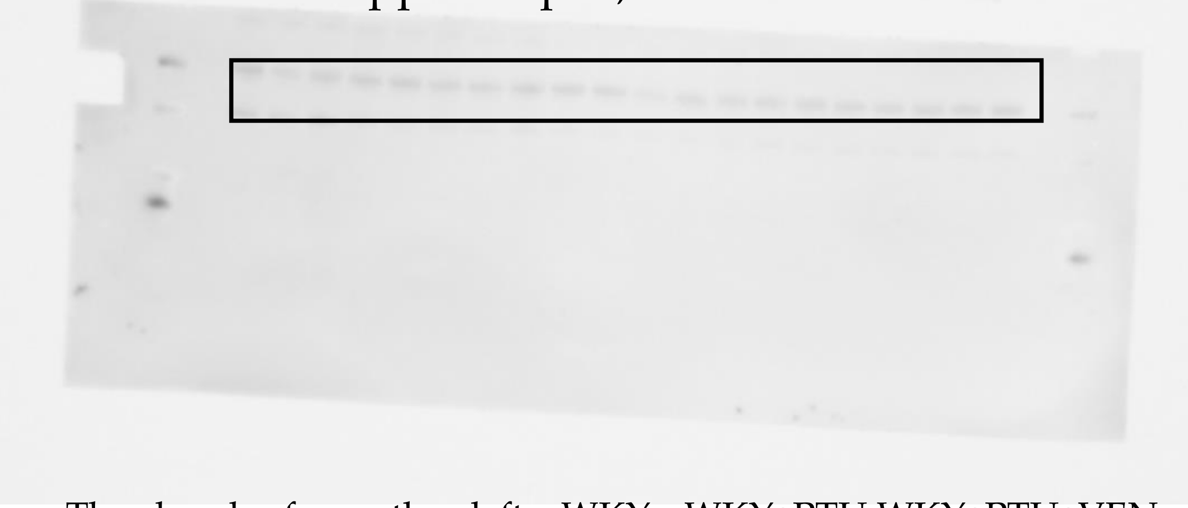

Vinculin as loading control to MCT4 Hippocampus, membrane 1 and 2

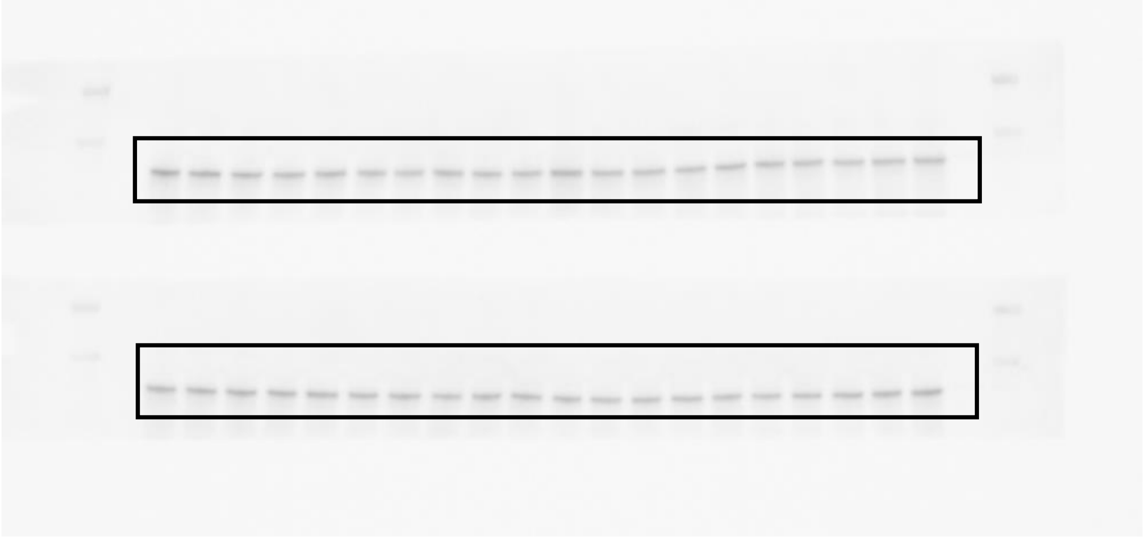

The bands from the left: WKY, WKY+PTU,WKY+PTU+VEN, WKY+PTU+T4, WKY+PTU+T4+VEN, WKY, WKY+PTU,WKY+PTU+VEN, WKY+PTU+T4, WKY+PTU+T4+VEN, WKY, WKY+PTU,WKY+PTU+VEN, WKY+PTU+T4, WKY+PTU+T4+VEN, WKY, WKY+PTU,WKY+PTU+VEN, WKY+PTU+T4, WKY+PTU+T4+VEN

The results of the experiment are shown in the Table 1

HCAR1 Frontal cortex, membrane 1

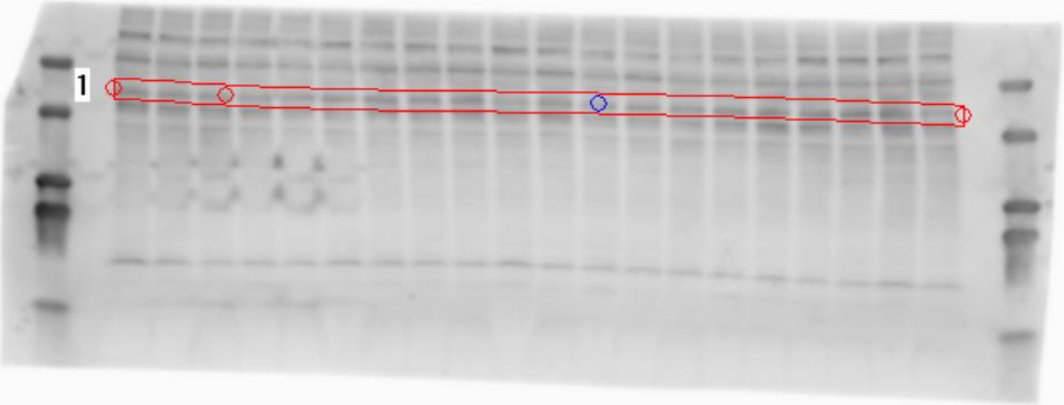

Vinculin as loading control to HCAR1 Frontal Cortex, membrane 1 and 2

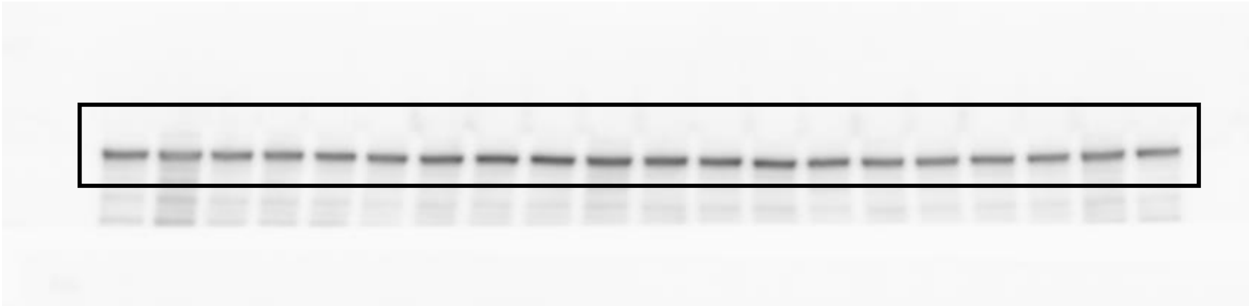

HCAR1 Frontal cortex, membrane 2

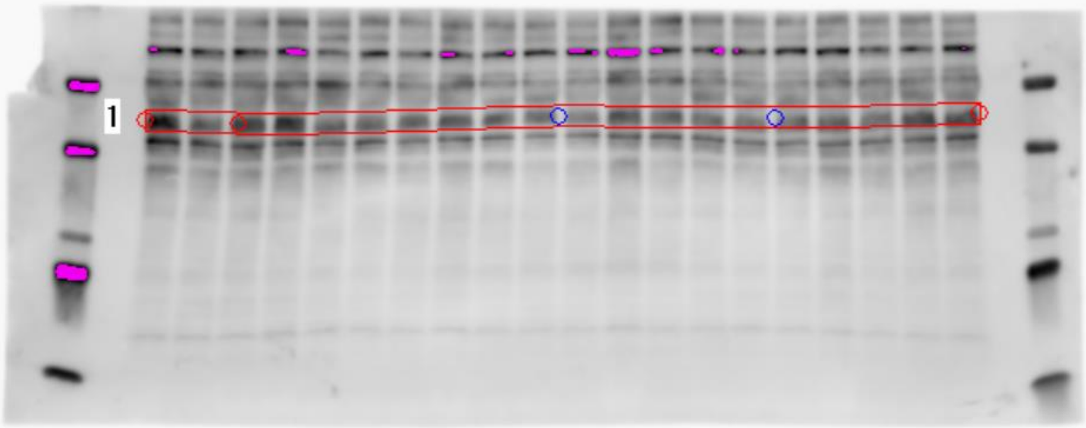

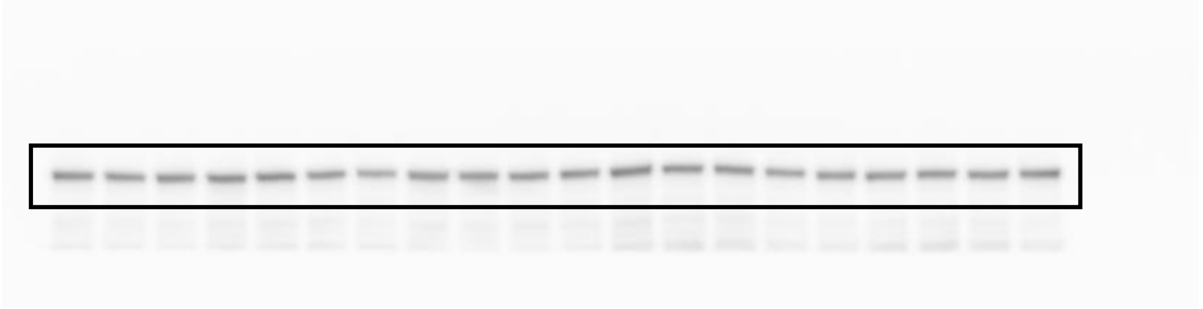

The bands from the left: WKY, WKY+PTU,WKY+PTU+VEN, WKY+PTU+T4, WKY+PTU+T4+VEN, WKY, WKY+PTU,WKY+PTU+VEN, WKY+PTU+T4, WKY+PTU+T4+VEN, WKY, WKY+PTU,WKY+PTU+VEN, WKY+PTU+T4, WKY+PTU+T4+VEN, WKY, WKY+PTU,WKY+PTU+VEN, WKY+PTU+T4, WKY+PTU+T4+VEN

The results of the experiment are shown in the Table 1

HCAR1 Hippocampus, membrane 1

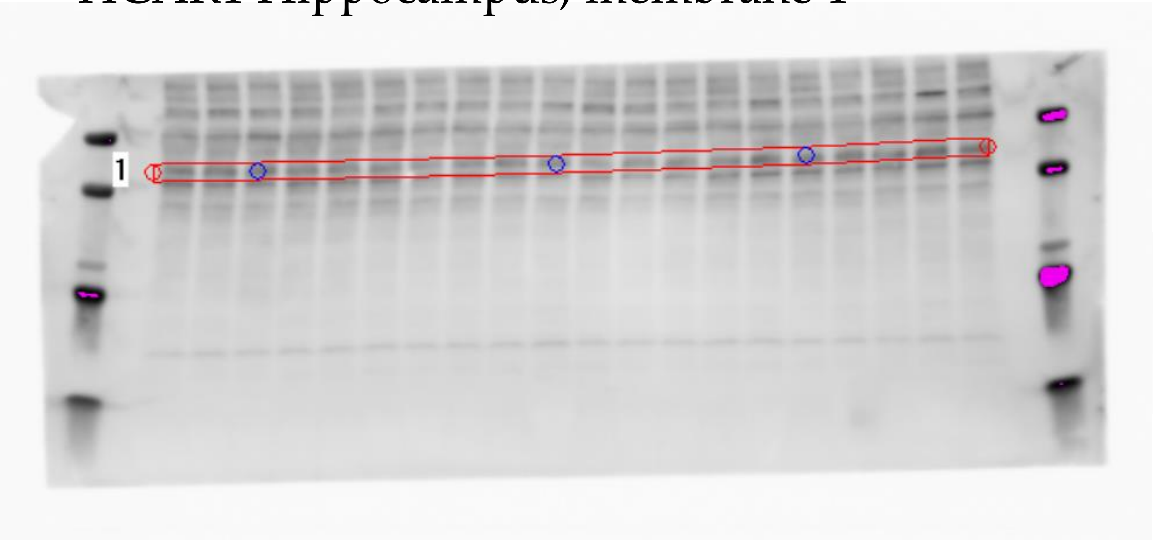

Vinculin as loading control to HCAR1 Hippocampus, membrane 1 and 2

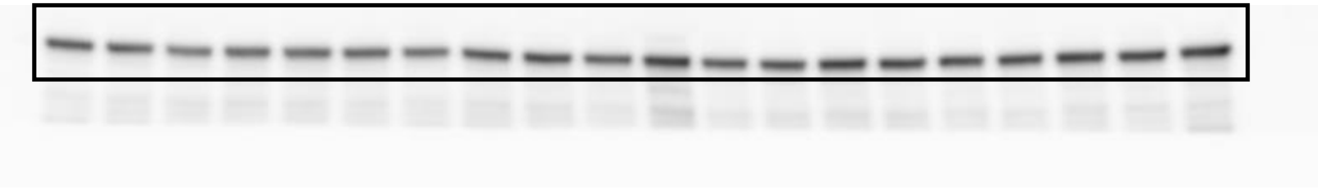

HCAR1 Hippocampus, membrane 2

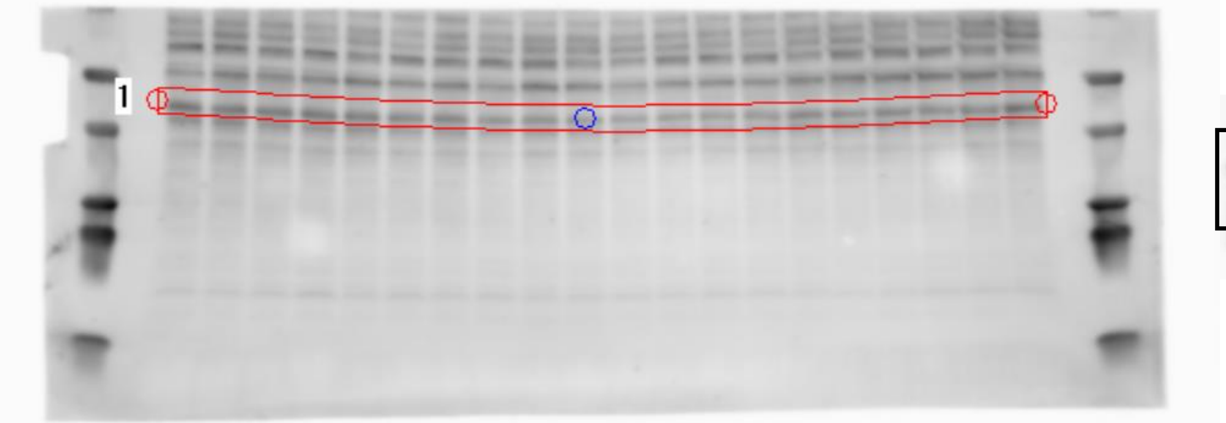

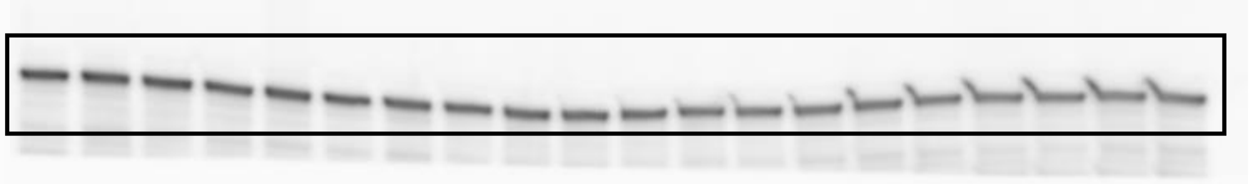

The bands from the left: WKY, WKY+PTU,WKY+PTU+VEN, WKY+PTU+T4, WKY+PTU+T4+VEN, WKY, WKY+PTU,WKY+PTU+VEN, WKY+PTU+T4, WKY+PTU+T4+VEN, WKY, WKY+PTU,WKY+PTU+VEN, WKY+PTU+T4, WKY+PTU+T4+VEN, WKY, WKY+PTU,WKY+PTU+VEN, WKY+PTU+T4, WKY+PTU+T4+VEN

The results of the experiment are shown in the Table 1

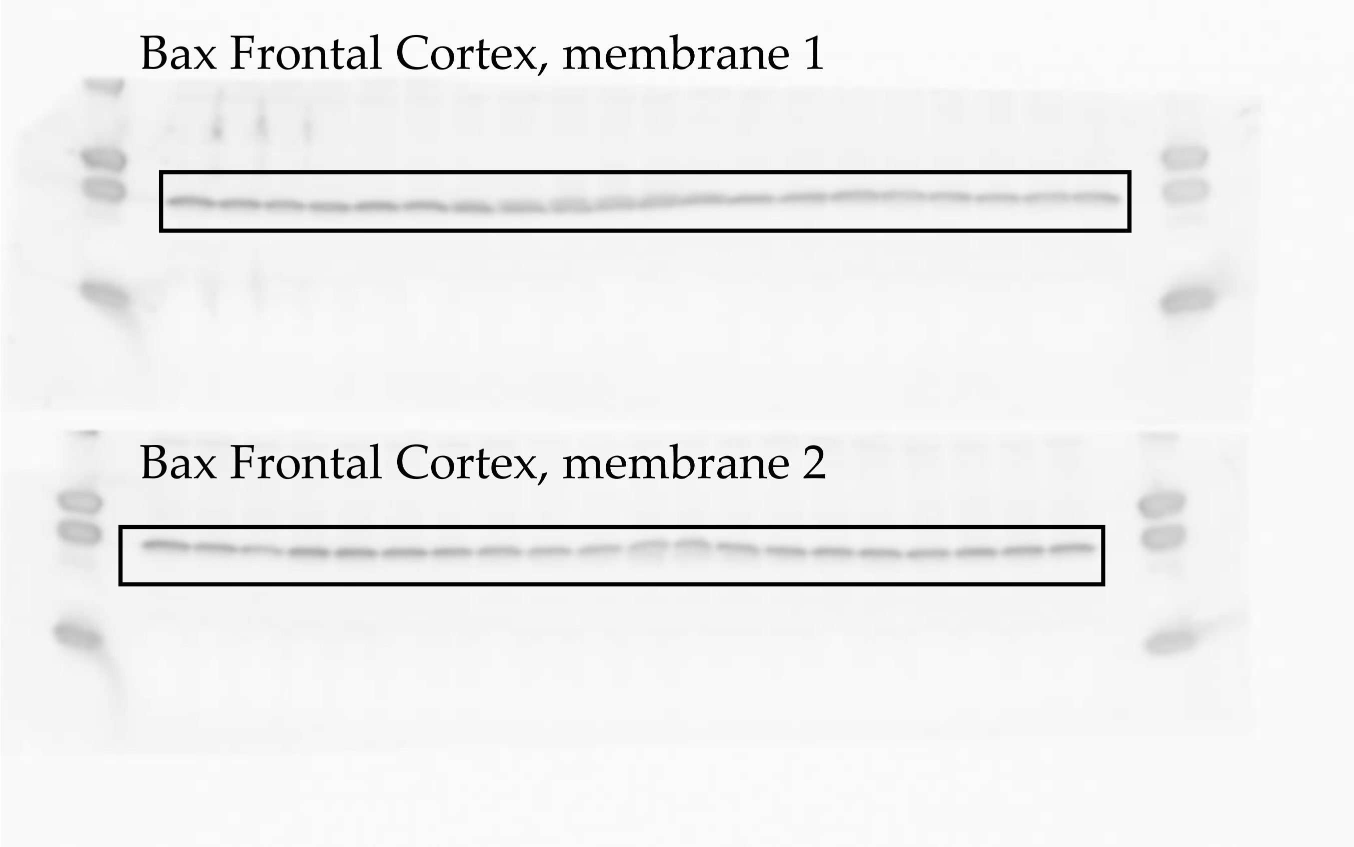

Vinculin as loading control to Bax Frontal Cortex, membrane 1 and 2

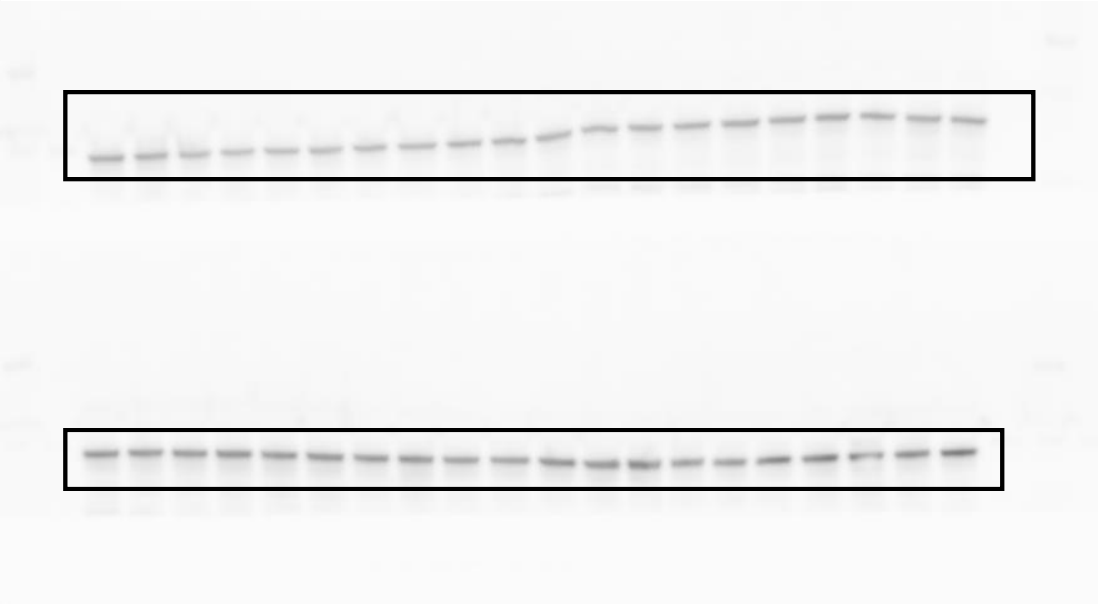

The bands from the left: WKY, WKY+PTU,WKY+PTU+VEN, WKY+PTU+T4, WKY+PTU+T4+VEN, WKY, WKY+PTU,WKY+PTU+VEN, WKY+PTU+T4, WKY+PTU+T4+VEN, WKY, WKY+PTU,WKY+PTU+VEN, WKY+PTU+T4, WKY+PTU+T4+VEN, WKY, WKY+PTU,WKY+PTU+VEN, WKY+PTU+T4, WKY+PTU+T4+VEN

The results of the experiment are shown in the Table 2

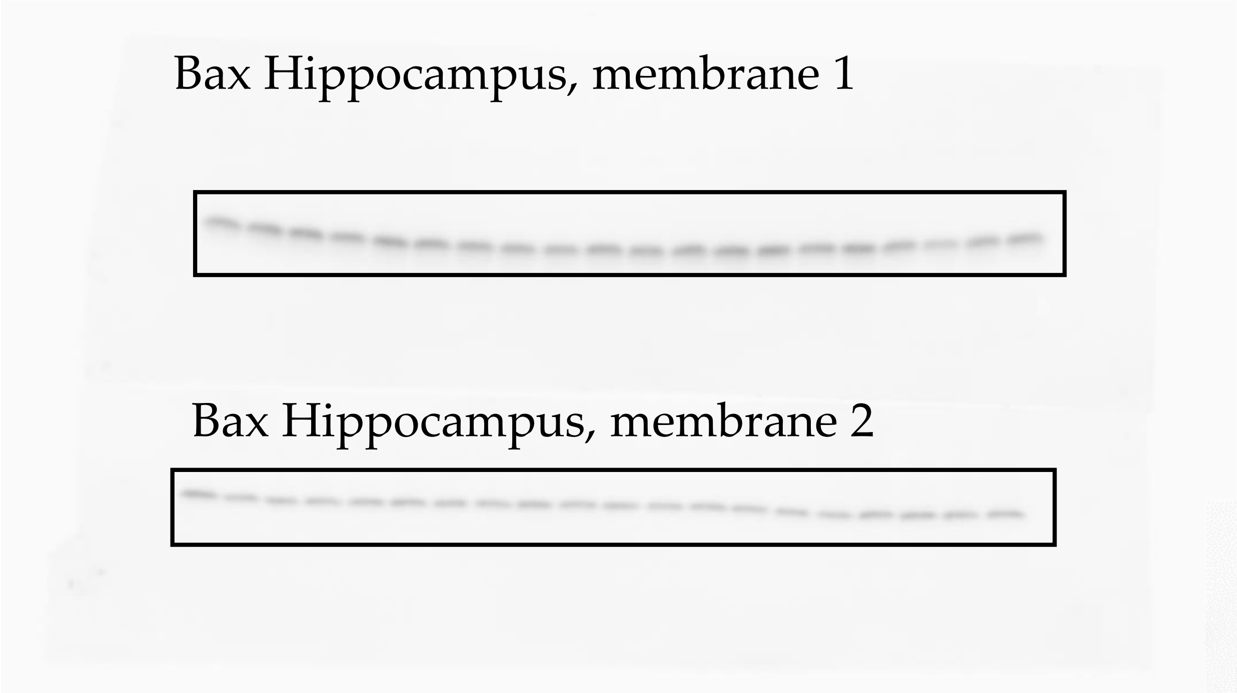

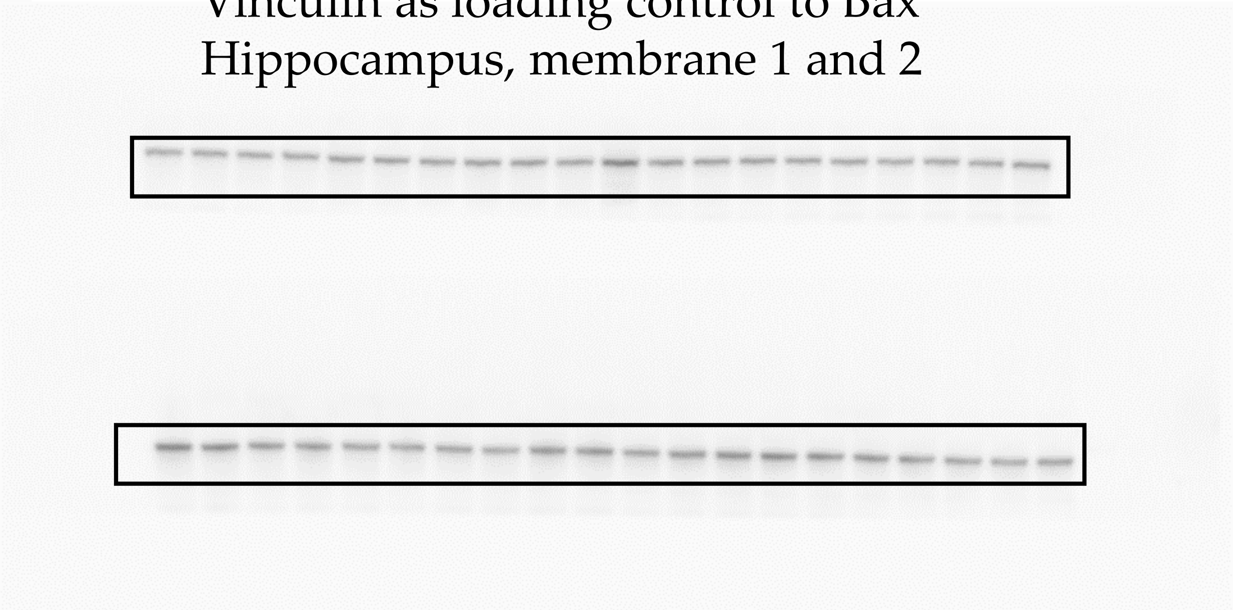

The bands from the left: WKY, WKY+PTU,WKY+PTU+VEN, WKY+PTU+T4, WKY+PTU+T4+VEN, WKY, WKY+PTU,WKY+PTU+VEN, WKY+PTU+T4, WKY+PTU+T4+VEN, WKY, WKY+PTU,WKY+PTU+VEN, WKY+PTU+T4, WKY+PTU+T4+VEN, WKY, WKY+PTU,WKY+PTU+VEN, WKY+PTU+T4, WKY+PTU+T4+VEN

The results of the experiment are shown in the Table 2

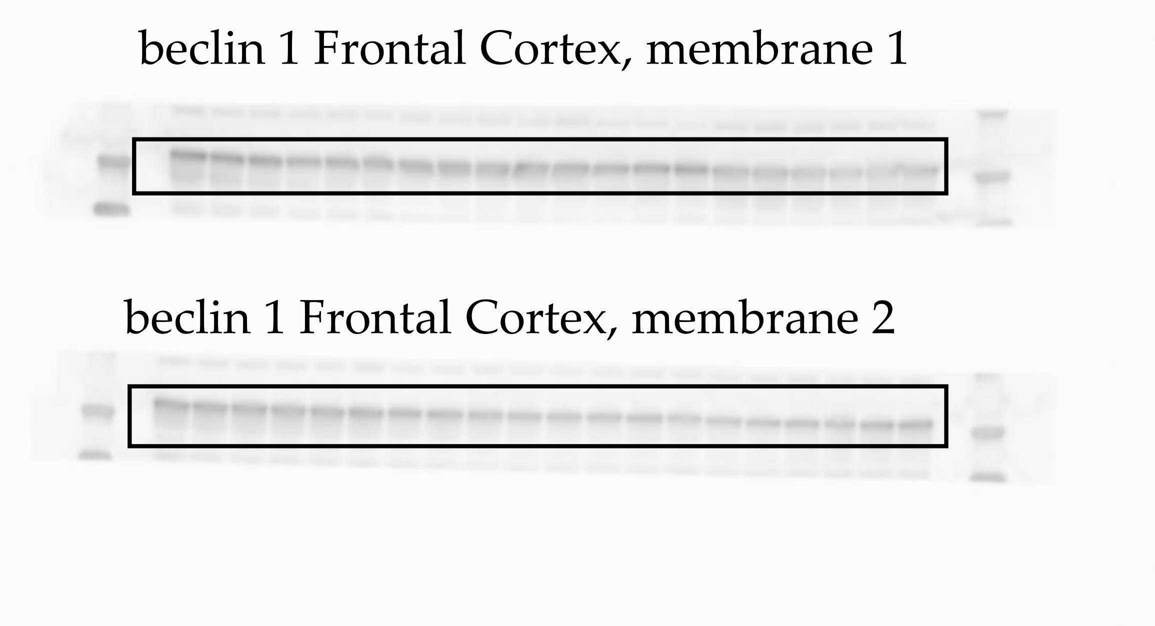

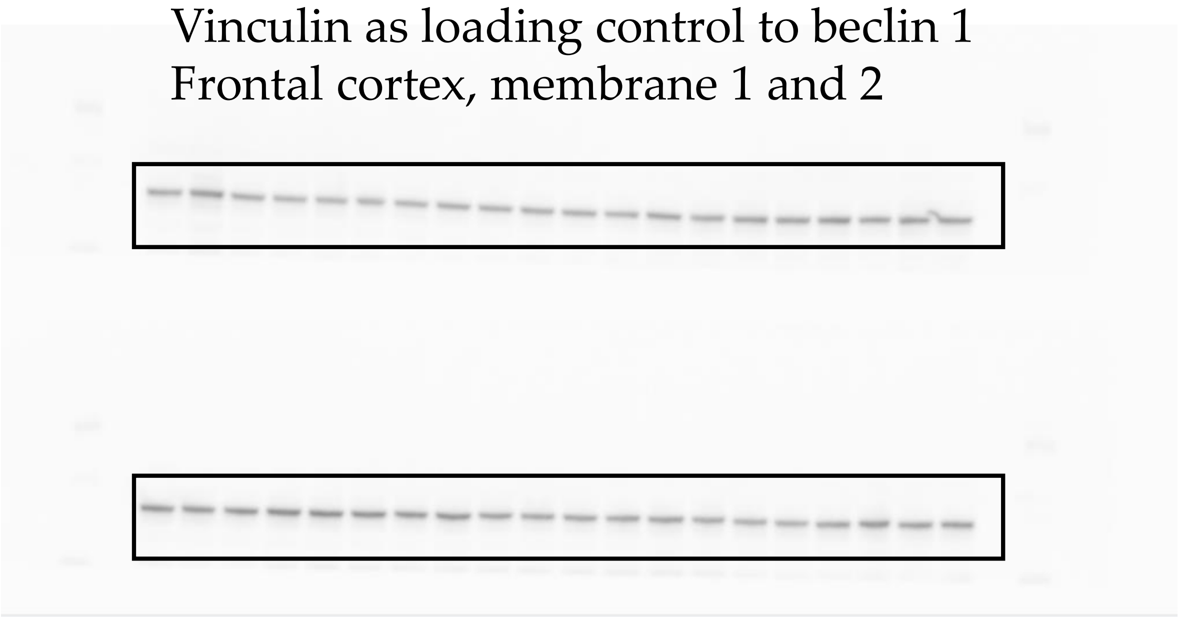

The bands from the left: WKY, WKY+PTU,WKY+PTU+VEN, WKY+PTU+T4, WKY+PTU+T4+VEN, WKY, WKY+PTU,WKY+PTU+VEN, WKY+PTU+T4, WKY+PTU+T4+VEN, WKY, WKY+PTU,WKY+PTU+VEN, WKY+PTU+T4, WKY+PTU+T4+VEN, WKY, WKY+PTU,WKY+PTU+VEN, WKY+PTU+T4, WKY+PTU+T4+VEN

The results of the experiment are shown in the Table 2

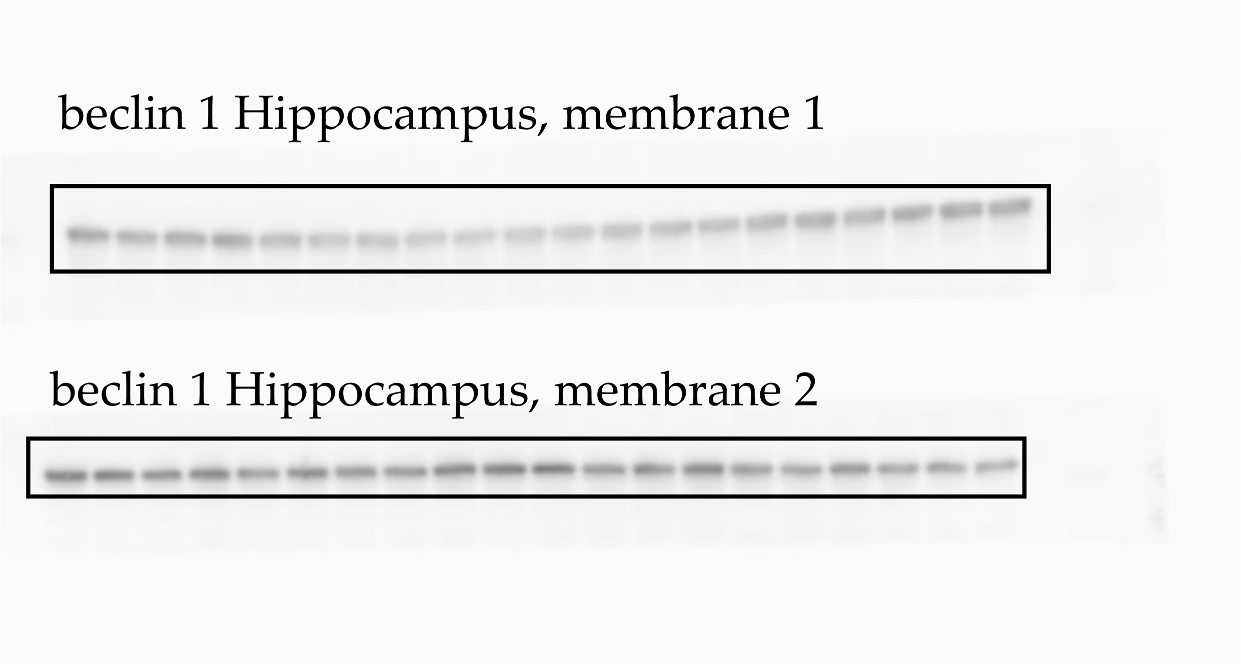

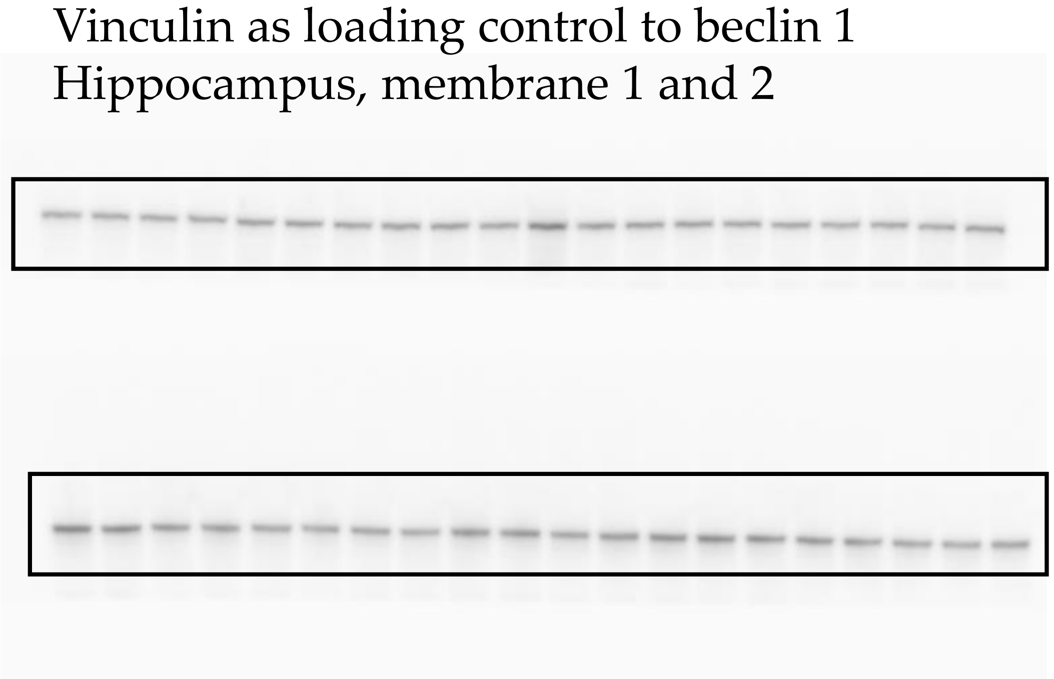

The bands from the left: WKY, WKY+PTU,WKY+PTU+VEN, WKY+PTU+T4, WKY+PTU+T4+VEN, WKY, WKY+PTU,WKY+PTU+VEN, WKY+PTU+T4, WKY+PTU+T4+VEN, WKY, WKY+PTU,WKY+PTU+VEN, WKY+PTU+T4, WKY+PTU+T4+VEN, WKY, WKY+PTU,WKY+PTU+VEN, WKY+PTU+T4, WKY+PTU+T4+VEN

The results of the experiment are shown in the Table 2

not all WB samples (presented on pages 1-18) were analyzed (poor quality or outliers were not analyzed)
